# Uncovering ecological state dynamics with hidden Markov models

**DOI:** 10.1111/ele.13610

**Published:** 2020-10-19

**Authors:** Brett T. McClintock, Roland Langrock, Olivier Gimenez, Emmanuelle Cam, David L. Borchers, Richard Glennie, Toby A. Patterson

**Affiliations:** ^1^ NOAA National Marine Fisheries Service Seattle WA USA; ^2^ Department of Business Administration and Economics Bielefeld University Bielefeld Germany; ^3^ CNRS Centre d'Ecologie Fonctionnelle et Evolutive Montpellier France; ^4^ Laboratoire des Sciences de l'Environnement Marin Institut Universitaire Européen de la Mer Univ. Brest CNRS, IRD Ifremer France; ^5^ School of Mathematics and Statistics University of St Andrews St Andrews UK; ^6^ CSIRO Oceans and Atmosphere Hobart Australia

**Keywords:** Behavioural ecology, community ecology, ecosystem ecology, hierarchical model, movement ecology, observation error, population ecology, state‐space model, time series

## Abstract

Ecological systems can often be characterised by changes among a finite set of underlying states pertaining to individuals, populations, communities or entire ecosystems through time. Owing to the inherent difficulty of empirical field studies, ecological state dynamics operating at any level of this hierarchy can often be unobservable or ‘hidden’. Ecologists must therefore often contend with incomplete or indirect observations that are somehow related to these underlying processes. By formally disentangling state and observation processes based on simple yet powerful mathematical properties that can be used to describe many ecological phenomena, hidden Markov models (HMMs) can facilitate inferences about complex system state dynamics that might otherwise be intractable. However, HMMs have only recently begun to gain traction within the broader ecological community. We provide a gentle introduction to HMMs, establish some common terminology, review the immense scope of HMMs for applied ecological research and provide a tutorial on implementation and interpretation. By illustrating how practitioners can use a simple conceptual template to customise HMMs for their specific systems of interest, revealing methodological links between existing applications, and highlighting some practical considerations and limitations of these approaches, our goal is to help establish HMMs as a fundamental inferential tool for ecologists.

## Graphical abstract

By formally disentangling state and observation processes based on simple yet powerful mathematical properties that can be used to describe many ecological phenomena, hidden Markov models (HMMs) can facilitate inferences about complex system state dynamics that might otherwise be intractable. We provide a gentle introduction to HMMs, establish some common terminology, review the immense scope of HMMs for applied ecological research, and provide a tutorial on some of the more technical aspects of HMM implementation and interpretation. By illustrating how practitioners can use a simple conceptual template to customise HMMs for their specific systems of interest, revealing methodological links between existing applications, and highlighting some practical considerations and limitations of these approaches, our goal is to help establish HMMs as a fundamental inferential tool for ecologists.

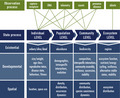

## INTRODUCTION

**Figure 1:**
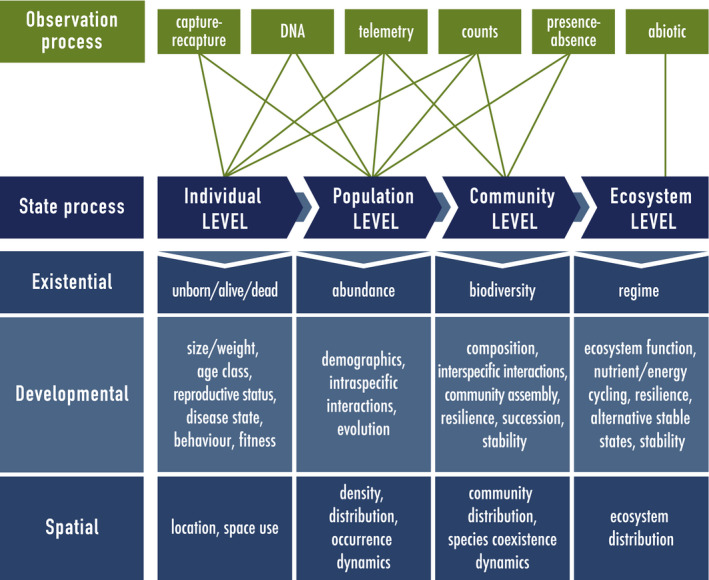
System state processes that can be difficult to observe directly, but can be uncovered from common ecological observation processes using hidden Markov models. The state process (blue) can pertain to any level within the ecological hierarchy (‘Individual’, ‘Population’, ‘Community’ or ‘Ecosystem’) and for convenience is categorised as primarily ‘Existential’, ‘Developmental’ or ‘Spatial’ in nature. The observation process (green) can provide information about state processes at different levels of the hierarchy (green lines) and includes capture–recapture, DNA sampling, animal‐borne telemetry, count surveys, presence–absence surveys and/or abiotic measurements. Observation and state processes from lower levels can be integrated for inferences at higher levels. For example, community‐level biodiversity data could be combined with environmental data to describe ecosystem‐level processes.

Ecological systems can often be characterised by changes among underlying system states through time. These state dynamics can pertain to individuals (e.g. birth, death), populations (e.g. increases, decreases), metapopulations (e.g. colonisation, extinction), communities (e.g. succession) or entire ecosystems (e.g. regime shifts). Gaining an understanding of state dynamics at each level of this hierarchy is a central goal of ecology and fundamental to studies of climate change, biodiversity, species distribution and density, habitat and patch selection, population dynamics, behaviour, evolution and many other phenomena (Begon *et al*., 2006). However, inferring ecological state dynamics is challenging for several reasons, including: (1) these complex systems often display nonlinear, non‐monotonic, non‐stationary and non‐Gaussian behaviour (Scheffer *et al*., 2001; Tucker and Anand, 2005; Wood, 2010; Pedersen *et al*., 2011a; Fasiolo *et al*., 2016); (2) changes in underlying states and dynamics can be rapid and drastic, but also gradual and more subtle (Beisner *et al*., 2003; Scheffer and Carpenter, 2003; Folke *et al*., 2004); and (3) the actual state of an ecological entity, be it an individual plant or animal, or a population or community, can often be difficult or impossible to observe directly (Martin *et al*., 2005; Kéry and Schmidt, 2008; Royle and Dorazio, 2008; Chen *et al*., 2013; Kellner and Swihart, 2014). Ecologists must therefore often contend with pieces of evidence believed to be informative of the state of an unobservable system at a particular point in time (see Fig. 1).

Whether for management, conservation or empirical testing of ecological theory, there is a need for inferential methods that seek to uncover the relationships between factors driving such systems, and thereby predict them in quantitative terms. Hidden Markov models (HMMs) constitute a class of statistical models that has rapidly gained prominence in ecology because they are able to accommodate complex structures that account for changes between unobservable system states (Ephraim and Merhav, 2002; Cappé *et al*., 2005; Zucchini *et al*., 2016). By simultaneously modelling two time series – one consisting of the underlying state dynamics and a second consisting of observations arising from the true state of the system – HMMs are able to detect state changes in noisy time‐dependent phenomena by formally disentangling the state and observation processes. For example, using HMMs and their variants:

- Historical regime shifts can be identified from reconstructed chronologies;
- Long‐term dynamics of populations, species, communities and ecosystems in changing environments can be inferred from dynamic biodiversity data;
- Species identity and biodiversity can be determined from environmental DNA (eDNA);
- Hidden evolutionary traits can be accounted for when assessing the drivers of diversification;
- Species occurrence can be linked to variation in habitat, population density, land use, host–pathogen dynamics or predator–prey interactions;
- Survival, dispersal, reproduction, disease status and habitat use can be inferred from capture–recapture time series;
- Animal movements can be classified into foraging, migrating or other modes for inferences about behaviour, activity budgets, resource selection and physiology; and
- Trade‐offs between dormancy and colonisation can be inferred from standing flora or fungal fruiting bodies.

The increasing popularity of HMMs has been fuelled by new and detailed data streams, such as those arising from modern remote sensing and geographic information systems (Viovy and Saint, 1994; Gao, 2002), eDNA (Bálint *et al*., 2018) and genetic sequencing (Hudson, 2008), as well as advances in computing power and user‐friendly software (Visser and Speenkenbrink, 2010). However, despite their utility and ubiquity in other fields such as finance (Bhar and Hamori, 2004), speech recognition (Rabiner, 1989) and bioinformatics (Durbin *et al*., 1998), the vast potential of HMMs for uncovering latent system dynamics from readily available data remains largely unrecognised by the broader ecological community. This is likely attributable to a tendency for the existing ecological literature to characterise HMMs as a subject‐specific tool reserved for a particular type of data rather than a general conceptual framework for probabilistic modelling of sequential data. This is also likely exacerbated by a tendency for HMMs to be applied and described quite differently across disciplines. Indeed, many ecologists may not recognise that some of the most well‐established inferential frameworks in population, community and movement ecology are in fact special cases of HMMs.

Catering to ecologists and non‐statisticians, we describe the structure and properties of HMMs (HIDDEN MARKOV MODELS), establish some common terminology (Table 1) and review case studies from the biological, ecological, genetics and statistical literature (ECOLOGICAL APPLICATIONS OF HIDDEN MARKOV MODELS). Central to our review and synthesis is a simple but flexible conceptual template that ecologists can use to customise HMMs for their specific systems of interest. In addition to highlighting new areas where HMMs may be particularly promising in ecology, we also demonstrate cases where these models have (perhaps unknowingly) already been used by ecologists for decades. We then identify some practical considerations, including implementation, software and potential challenges that practitioners may encounter when using HMMs (IMPLEMENTATION, CHALLENGES AND PITFALLS). Using an illustrative example, we provide a step‐by‐step tutorial on some of the more technical aspects of HMM implementation in the Supplementary Tutorial. The overall aim of our review is thus to provide a synthesis of the various ways in which HMMs can be used, reveal methodological links between existing applications and thereby establish HMMs as a fundamental inferential tool for ecologists working with sequential data.

**Table 1:** Glossary

Term	Definition	Synonyms
Conditional independence property	Assumption made for the state‐dependent process: conditional on the state at time *t*, the observation at time *t* is independent of all other observations and states	
Forward algorithm	Recursive scheme for updating the likelihood and state probabilities of an HMM through time	Filtering
Forward–backward algorithm	Recursive scheme for calculating state probabilities for any point in time: $\mathrm{Pr}(S_{t}=i|x_{1},\ldots ,x_{T})$	Local state decoding; smoothing
Hidden Markov model (HMM)	A special class of state‐space model with a finite number of hidden states that typically assumes some form of the Markov property and the conditional independence property	Dependent mixture model; latent Markov model; Markov‐switching model; regime‐switching model; state‐switching model; multi‐state model
Initial distribution $\left(\delta \right)$	The probability of being in any of the N states at the start of the sequence: $\delta =\left(\mathrm{Pr}\left(S_{1}=1\right),\ldots ,\mathrm{Pr}\left(S_{1}=N\right)\right)$	Initial probabilities; prior probabilities
Markov property	Assumption made for the state process: $\mathrm{Pr}\left(S_{t+1}|S_{t},\;S_{t‐1},\ldots \right)=\mathrm{Pr}\left(S_{t+1}|S_{t}\right)$ (‘conditional on the present, the future is independent of the past’)	Memoryless property
Sojourn time	The amount of time spent in a state before switching to another state	Dwell time; occupancy time
State process $\left(S_{t}\right)$	Unobserved, serially correlated sequence of states describing how the system evolves over time: $S_{t}\in \left\{1,\ldots ,N\right\}$ for t=1,…,T	Hidden/latent process; system process
State transition probability $\left(\gamma_{\mathrm{ij}}\right)$	The probability of switching from state i at time t to state j at time t+1, $\gamma_{\mathrm{ij}}=\mathrm{Pr}\left(S_{t+1}=j|S_{t}=i\right)$, usually represented as an N×N transition probability matrix $\left(\Gamma \right)$	
State‐dependent distribution $\left(f\left(x_{t}|S_{t}=i\right)\right)$	Probability distribution of an observation $x_{t}$ conditional on a particular state being active at time t, usually from some parametric class (e.g. categorical, Poisson, normal) and represented as an N×N diagonal matrix $P\left(x_{t}\right)=\text{diag}\left(f\left(x_{t}|S_{t}=1\right),\ldots ,f\left(x_{t}|S_{t}=N\right)\right)$	Emission distribution; measurement model; observation distribution; output distribution; response distribution
State‐dependent process $\left(X_{t}\right)$	The observed process within an HMM, which is assumed to be driven by the underlying unobserved state process	Observation process
State‐space model	A conditionally specified hierarchical model consisting of two linked stochastic processes, a latent system process model and an observation process model	
Viterbi algorithm	Recursive scheme for finding the sequence of states which is most likely to have given rise to the observed sequence	Global state decoding

## HIDDEN MARKOV MODELS

We begin by providing a gentle introduction to HMMs, including model formulation, inference and extensions. Although we have endeavoured to minimise technical material and provide illustrative examples wherever possible, we assume the reader has at least some basic understanding of linear algebra concepts such as matrix multiplication and diagonal matrices (e.g. see Appendix A in Caswell, 2001) and probability theory concepts such as uncertainty, random variables and probability distributions (Gotelli and Ellison, 2013, Chapters 1–2).

### Basic model formulation

Hidden Markov models (HMMs) are a class of statistical models for sequential data, in most instances related to systems evolving over time. The system of interest is modelled using a *state process* (or *system process*; Table 1), which evolves dynamically such that future states depend on the current state. Many ecological phenomena can naturally be described by such a process (Fig. 1). In an HMM, the state process is not directly observed – it is a ‘hidden’ (or ‘latent’) variable. Instead, observations are made of a *state‐dependent process* (or *observation process*) that is driven by the underlying state process. As a result, the observations can be regarded as noisy measurements of the system states of interest, but they are typically insufficient to precisely determine the state. Mathematically, an HMM is composed of two sequences:

Box 1 Where do HMMs reside in the taxonomic zoo of latent variable models? 1 Latent state (or latent variable) models come in many different forms, with a particular variant often evolving its own nomenclature, notation and jargon that can be confusing for non‐specialists. Here we use broad and non‐technical strokes to differentiate the HMM from its close relatives in the taxonomy of latent state models, with the aim to more clearly position HMMs relative to alternative modelling frameworks. Above all, these models are united by assuming *latent states* – a fundamental property of the system being modelled that is either partially, or completely, unobservable. They also tend to make a clear distinction between an observation process model – describing noise in the data – and the hidden state process model – describing the underlying patterns and dynamics of interest.

The umbrella terms mixed effects, multilevel or hierarchical models (e.g. Skrondal and Rabe‐Hesketh, 2004; Gelman and Hill, 2006; Royle and Dorazio, 2008; Lee and Song, 2012) typically include the most widely known types of latent variable models (e.g. Clogg, 1995). These often treat latent variables as random effects assumed to arise from a distribution as structural elements of a hierarchical statistical model. There is therefore not only random variation in the observations, but also in the parameters of the model itself. While there are special cases and generalisations that are not so easily classified, a simplified taxonomy for a subset of hierarchical latent variable models can be based on the structural dependence in the hidden state process and whether the state space of this hidden process is discrete (i.e. taking on finitely many values) or continuous:

**Table:** 

	State space	
	Continuous	Discrete
Temporal dependence	State‐space model	Hidden Markov model
Temporal independence	Continuous mixture model	Finite mixture model

Latent variable models with a continuous state space and no temporal dependence in the hidden state process fall under the broad class of *continuous mixture models* (e.g. Lindsay, 1995), with ecological applications including the modelling of closed population abundance (Royle, 2004), disease prevalence (Calabrese *et al*., 2011) and species distribution (Ovaskainen *et al*., 2017). *State‐space models* (SSMs) are a special class of latent variable model where the observation process is conditionally specified by a (typically continuous) hidden state process with temporal dependence (e.g. Durbin and Koopman, 2012; Auger‐Méthé *et al*., 2020), with applications including population dynamics (Schnute, 1994; Wang, 2007; Tavecchia *et al*., 2009; Newman *et al*., 2014), disease dynamics (Rohani and King, 2010; Cooch *et al*., 2012) and animal movement (Patterson *et al*., 2008; Hooten *et al*., 2017; Patterson *et al*., 2017). An HMM is a special class of SSM where the state space is finite (see ECOLOGICAL APPLICATIONS OF HIDDEN MARKOV MODELS for many ecological examples). *Finite mixture models* (e.g. Frühwirth‐Schnatter, 2006) assume the state space is finite with no temporal dependence in the hidden state process (e.g. the latent states are non‐Markov or do not change over time), with examples including static species occurrence (MacKenzie *et al*., 2002), closed population capture–recapture (Pledger, 2000) and species distribution (Pledger and Arnold, 2014) models. HMMs and SSMs can therefore be regarded as specific variations of a hierarchical model with serial dependence, where the random effects vary over time. Furthermore, an HMM can be viewed as a discrete version of a SSM or a time‐dependent version of a finite mixture model.

It is important to note that things are not quite as simple as depicted above. For example, while an SSM with discrete latent variables can encompass features of an HMM (Jonsen *et al*., 2005), an SSM with a finite state space is not necessarily an HMM. An HMM might include continuous random effects on its parameters or a state‐dependent observation distribution specified as a finite mixture (Altman, 2007). If the number of states becomes very large in an HMM, then it can become a discrete approximation of an SSM with a continuous state space (Besbeas and Morgan, 2019). In the Extensions section and the Challenges and Pitfalls section, we consider circumstances where application of a standard HMM is not supported and other approaches or extensions might be required. [Correction added on 10 November 2020, after first online publication: Box 1 has been relocated to page 5.]

Box 2 To HMM, or not to HMM, that is the question 1 The structure of a statistical model should be congruent with the data‐generating process in question. HMMs are neither a panacea nor a black box – the appropriateness and feasibility of a particular model will be case‐dependent and requires careful consideration. In determining if HMMs are appropriate for describing a particular system, one must consider two questions:

1. **Do the hidden state dynamics display time dependence which can be represented using Markov chains?** If the current system state is not related to the previous state(s), then a latent variable model without time dependence should be considered (see Box 1). Diagnostics examining temporal patterns in residuals (Li, 2003) can help to empirically determine if the assumptions of conditional independence and Markovity are sufficient (see Supplementary Tutorial). When the first‐order Markov assumption may not be appropriate for the state process, one can further ask the question: *can system memory be adequately approximated while preserving Markovity?* Faithful representation of system memory may require the inclusion of informative covariates or more complex time dependence structures, and it is possible to expand HMMs to higher order Markovian or semi‐Markovian dependence (Zucchini *et al*., 2016, Chapter 12). While modelling this higher order temporal dependence is sometimes preferable (Hestbeck *et al*., 1991), it is more complex and thus less widely used. General time‐series modelling often captures complex dependence structures using autoregressive processes (Durbin and Koopman, 2012, Chapter 3), and more complicated variations of HMMs can capture some of these features (Lawler *et al*., 2019). However, other latent variable approaches will often be better suited for more complex temporal dependence structures. There is no foolproof or automatic way to make this determination, and we must typically rely on residual diagnostics (Li, 2003; Zucchini *et al*., 2016, Chapter 6) and expert knowledge of the system dynamics.
2. **Can the system be well described by a feasibly finite set of latent states?** Our review highlights a wide range of ecological scenarios where the possible states of the system of interest form (or can be approximated by) a finite set. The number of parameters and the computational burden of an HMM can become large with increases in state dimension, and this can be of particular concern when the finite set of states is a coarser approximation of a finer discrete space (e.g. population abundance) or a continuous space (e.g. spatial location). Such approximations have strengths and weaknesses. When used as discrete approximations to state‐space models with continuous support (see Box 1), HMMs can be useful when arbitrary constraints on the state space are required (e.g. restricting aquatic organisms to location states off land) or when combining both discrete and continuous state processes. However, an HMM for a large number of states with a fully parameterised transition probability matrix – where transitions between any of the states are possible – will be computationally expensive, perhaps prohibitively so. Systems with large state spaces can often be approximated by an HMM when transitions between states are local – where transitions can only occur between neighbouring states – and the transition probabilities therefore include a relatively small number of parameters that describe this local behaviour. For example, Thygesen *et al*. (2009), Pedersen *et al*. (2011b), and Glennie *et al*. (2019) use these properties of sparsity to make an HMM approach computationally efficient for very large state spaces. In short, large numbers of states do not necessarily prohibit application of an HMM; this is dependent on the computer resources available and the properties of the state process. Alternatively, it is possible to reduce the size of an infeasible state space by making a coarser approximation (e.g. binning abundance states together into larger states; Zucchini *et al*., 2016, pp. 162–163; Besbeas and Morgan, 2019). Appropriateness will depend on the sensitivity of the inference to the precise value of the state process and is best investigated by varying the coarseness of the approximation. If the set of states is too coarse‐grained, approximation might lead to spurious inference about the latent states. For example, coarse‐graining could result in masking or misclassification of meaningfully distinct states. The decision of the appropriate number of states can be challenging; there is again no foolproof or automatic way to determine this, and we must usually rely on expert knowledge of the specific system of interest. When the finite state space of an HMM is infeasible or inappropriate, it will often be better to consider other approaches (e.g. Patterson *et al*., 2008; Cooch *et al*., 2012; Patterson *et al*., 2017; Auger‐Méthé *et al*., 2020).

- An observed state‐dependent process $X_{1},X_{2},\ldots ,X_{T}$; and
- An unobserved (hidden) state process $S_{1},S_{2},\ldots ,S_{T}$.

In most applications, the indices refer to observations made over time at a regular sampling interval (e.g. daily or annual rainfall measurements), but they can also refer to position (e.g. in a sequence of DNA; Henderson *et al*., 1997; Eddy, 2004) or order (e.g. in a sequence of marine mammal dives; DeRuiter *et al*., 2017). HMMs can also be formulated in continuous time (Jackson *et al*., 2003; Amoros *et al*., 2019), but these models have tended to be less frequently applied in ecology (but see Langrock *et al*., 2013; Choquet *et al*., 2017; Olajos *et al*., 2018). Among the many HMM formulations of relevance to ecology that we highlight in ECOLOGICAL APPLICATIONS OF HIDDEN MARKOV MODELS, some example observation sequences $\left(X_{1},\ldots ,X_{T}\right)$ and underlying states $\left(S_{1},\ldots ,S_{T}\right)$ include:

- $X_{t}=$ Observation of feeding/not feeding, with underlying state $S_{t}=$ Hungry or sated;
- $X_{t}=$ Count of individuals, with underlying state $S_{t}=$ True population abundance; and
- $X_{t}=$ Daily rainfall measurement, with underlying state $S_{t}=$ Wet or dry season.

**Figure 2:**
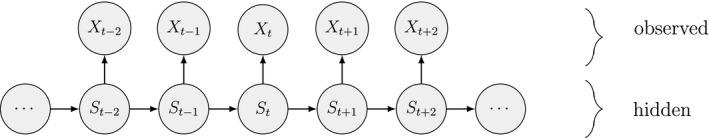
Dependence structure of a basic hidden Markov model, with an observed sequence $X_{1},\ldots ,X_{T}$ arising from an unobserved sequence of underlying states $S_{1},\ldots ,S_{T}$.

Unlike the larger class of *state‐space models* (see Box 1), the state process within an HMM can take on only finitely many possible values: $S_{t}\in \left\{1,\ldots ,N\right\}$ for t=1,…,T. The basic HMM formulation further involves two key dependence assumptions: (1) the probability of a particular state being active at any time t is completely determined by the state active at time t‐1 (the so‐called *Markov property*); and (2) the probability distribution of an observation at any time t is completely determined by the state active at time t (Fig. 2). The latter assumption is a *conditional independence property*, as this implies that $X_{t}$ is conditionally independent of past and future observations, given $S_{t}$. Whether these simplifying assumptions can faithfully characterise the underlying dynamics for the system of interest must be carefully considered (see Challenges and pitfalls).

As a consequence of these assumptions, HMMs generally facilitate model building and computation that might otherwise be intractable. A basic N‐state HMM that formally distinguishes the state and observation processes can be fully specified by the following three components: (1) the *initial distribution*, $\delta =\left(\mathrm{Pr}\left(S_{1}=1\right),\ldots ,\mathrm{Pr}\left(S_{1}=N\right)\right)$, specifying the probabilities of being in each state at the start of the sequence; (2) the *state transition probabilities*, $\gamma_{\mathrm{ij}}=\mathrm{Pr}\left(S_{t+1}=j|S_{t}=i\right)$, specifying the probability of switching from state i at time t to state j at time t+1 and usually represented as an N×N state transition probability matrix:

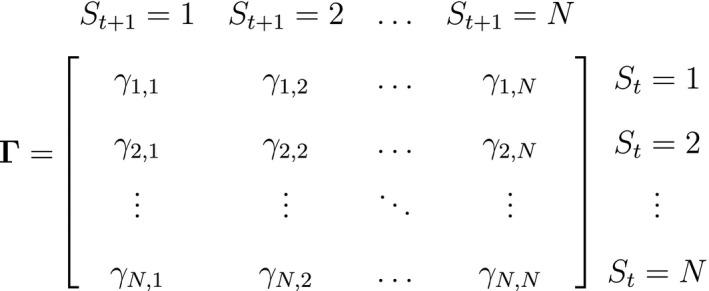

where $\sum_{j=1}^{N}\gamma_{ij}=1$; and 3) the *state‐dependent distributions*, $f(x_{t}|S_{t}=i)$, specifying the probability distribution of an observation $x_{t}$ conditional on the state at time t and usually represented as an N×N diagonal matrix:

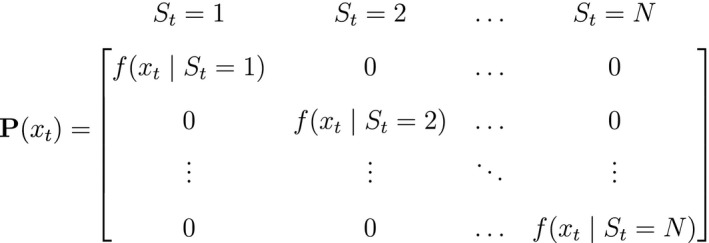

or, equivalently, $P\left(x_{t}\right)=\text{diag}\left(f\left(x_{t}|S_{t}=1\right),\ldots ,f\left(x_{t}|S_{t}=N\right)\right)$ for computational purposes (see Inference). These distributions can pertain to discrete or continuous observations and are generally chosen from an appropriate distributional family. For example, behavioural observation $X_{t}\in \left\{\text{feeding},\text{not}\text{feeding}\right\}$ could be modelled using a categorical distribution (MacDonald and Raubenheimer, 1995), count $X_{t}\in \left\{0,1,2,\ldots \right\}$ using a non‐negative discrete distribution (e.g. Poisson; Besbeas and Morgan, 2019, and measurement $X_{t}\in \left[0,\infty \right)$ using a non‐negative continuous distribution (e.g. zero‐inflated exponential; Woolhiser and Roldan, 1982). After specifying δ, Γ and $P\left(x_{t}\right)$ in terms of the particular system of interest, one can proceed to drawing inferences about unobservable state dynamics from the observation process.

We note that Markov models (Grewal *et al*., 2019) are commonly used for inferring community‐ or ecosystem‐level dynamics (Waggoner and Stephens, 1970; Wootton, 2001; Tucker and Anand, 2005; Breininger *et al*., 2010) and providing measures of stability, resilience or persistence (Li, 1995; Pawlowski and McCord, 2009; Zweig *et al*., 2020), especially in systems composed of sessile organisms such as plant (Horn, 1975; van Hulst, 1979; Usher, 1981; Talluto *et al*., 2017, but see Chen *et al*., 2013) or benthic communities (Tanner *et al*., 1994; Hill *et al*., 2004; Lowe *et al*., 2011). A Markov model can simply be viewed as an HMM where it is assumed that the state process is perfectly observed, that is, $X_{t}=S_{t}$ with $P\left(x_{t}\right)$ a matrix with entry one in row $s_{t}$, column $s_{t}$, and otherwise zeros. For example, patch dynamics HMMs (MacKenzie *et al*., 2003) are simply generalisations of well‐known Markov models for patch dynamics (Hanski, 1994; Moilanen, 1999) for cases when presence–absence data are subject to imperfect detection. Likewise, any Markov model can naturally be embedded as the state process within an HMM for less observable phenomena.

### Inference

In addition to the ease with which a wide variety of ecological state and observation process models can be specified (see ECOLOGICAL APPLICATIONS OF HIDDEN MARKOV MODELS), a key strength of the HMM framework is that efficient recursive algorithms are available for conducting statistical inference. Here we will briefly outline some of the most common inferential techniques for HMMs, but motivated readers can find additional technical material and a worked example on model fitting, assessment and interpretation in the Supplementary Tutorial. Using the *forward algorithm* (also known as *filtering*), the likelihood $L(\theta |x_{1},\ldots ,x_{T})$ as a function of the unknown parameters $\left(\theta \right)$ given the observation sequence $\left(x_{1},\ldots ,x_{T}\right)$ can be calculated at a computational cost that is (only) linear in T. The parameter vector θ, which is to be estimated, contains any unknown parameters embedded in the three model‐defining components δ, Γ and $P\left(x_{t}\right)$. Made possible by the relatively simple dependence structure of an HMM, the forward algorithm traverses along the time series, updating the likelihood step‐by‐step while retaining information on the probabilities of being in the different states (Zucchini *et al*., 2016, pp. 37–39). Application of the forward algorithm is equivalent to evaluating the likelihood using a simple matrix product expression,

(1) $$L(\theta |x_{1},...,x_{T})=\delta P\left(x_{1}\right)\Gamma P\left(x_{2}\right)\cdots \Gamma P\left(x_{T‐1}\right)\Gamma P\left(x_{T}\right)1,$$

where **1** is a column vector of ones (see Supplementary Tutorial for technical derivation).

In practice, the main challenge when working with HMMs tends to be the estimation of the model parameters. The two main strategies for fitting an HMM are numerical maximisation of the likelihood (Myung, 2003; Zucchini *et al*., 2016) or Bayesian inference (Ellison, 2004; Gelman *et al*., 2004) using Markov chain Monte Carlo (MCMC) sampling (Brooks *et al*., 2011). The former seeks to identify the parameter values that maximise the likelihood function (i.e. the maximum likelihood estimates $\hat{\theta }$), whereas the latter yields a sample from the posterior distribution of the parameters (Ellison, 2004). Specifically for the maximum likelihood (ML) approach, the forward algorithm makes it possible to use standard optimisation methods (Fletcher, 2013) to directly numerically maximise the likelihood (eqn 1). An alternative ML approach is to employ an expectation–maximisation (EM) algorithm that uses similar recursive techniques to iterate between *state decoding* and updating the parameter vector until convergence (Rabiner, 1989). For MCMC, many different strategies can be used, but these tend to differ in appropriateness and efficiency in a manner that can strongly depend on the specific model and data at hand (Gilks *et al*., 1996; Gelman *et al*., 2004; Brooks *et al*., 2011; Robert and Casella, 2004).

The forward algorithm and similar recursive techniques can further be used for forecasting and state decoding, as well as to conduct formal model checking using pseudo‐residuals (Zucchini *et al*., 2016, Chapters 5 & 6). State decoding is usually accomplished using the *Viterbi algorithm* or the *forward–backward algorithm* (also known as *smoothing*), which respectively identify the most likely sequence of states or the probability of each state at any time t, conditional on the observations. Fortunately, practitioners can often use existing software for most aspects of HMM‐based data analyses and need not dwell on many of the more technical details of implementation (see IMPLEMENTATION, CHALLENGES AND PITFALLS and Supplementary Tutorial).

To illustrate some of the basic mechanics, we use a simple example based on observations of the feeding behaviour of a blue whale (*Balaenoptera musculus*; cf. DeRuiter *et al*., 2017). Suppose we assume that observations of the number of feeding lunges performed in each of T=53 consecutive dives ($X_{t}\in \left\{0,1,2,\ldots \right\}$ for t=1,…,T) arise from N=2 states of feeding activity. Building on Fig. 2, we could for example have:

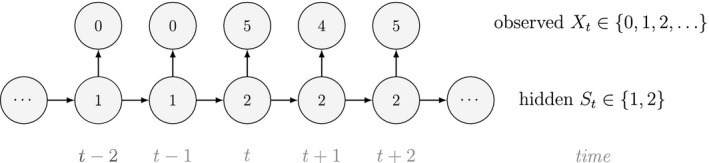

**Figure 3:**
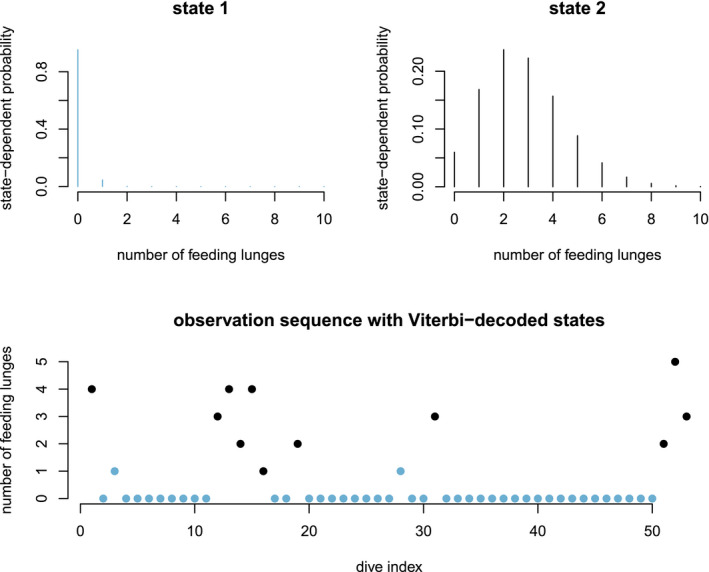
Estimated state‐dependent distributions (top row) and Viterbi‐decoded states from a two‐state HMM fitted to counts of feeding lunges performed by a blue whale during a sequence of T=53 consecutive dives. Here the most likely state sequence identifies periods of ‘low’ (state 1; blue) and ‘high’ (state 2; black) feeding activity.

Fig. 3 displays the results for this simple two‐state HMM assuming Poisson state‐dependent (observation) distributions, $X_{t}|S_{t}=i∼\text{Poisson}\left(\lambda_{i}\right)$ for $i\in \left\{1,2\right\}$, when fitted to the full observation sequence via direct numerical maximisation of eqn 1. The rates of the state‐dependent distributions were estimated as ${\hat{\lambda }}_{1}=0.05$ and ${\hat{\lambda }}_{2}=2.82$, suggesting states 1 and 2 correspond to ‘low’ and ‘high’ feeding activity respectively. The estimated state transition probability matrix,

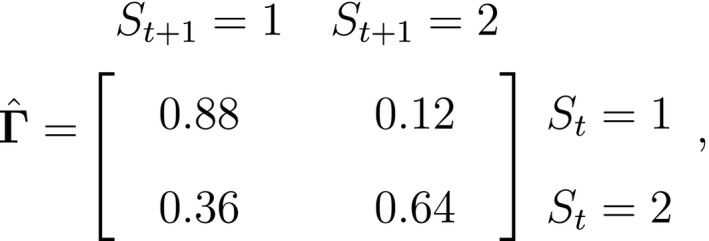

suggests interspersed bouts of ‘low’ and ‘high’ feeding activity, but with bouts of ‘high’ activity tending to span fewer dives. The estimated initial distribution $\hat{\delta }=\left(0.75,0.25\right)$ suggests this individual was more likely to have been in the ‘low’ activity state at the start of the sequence. Most ecological applications of HMMs involve more complex inferences related to specific hypotheses about system state dynamics, and a great strength of the HMM framework is the relative ease with which the basic model formulation can be modified to describe a wide variety of processes (Zucchini *et al*., 2016, Chapters 9–13). Next we highlight some extensions that we consider to be highly relevant in ecological research.

### Extensions

**Figure 4:**
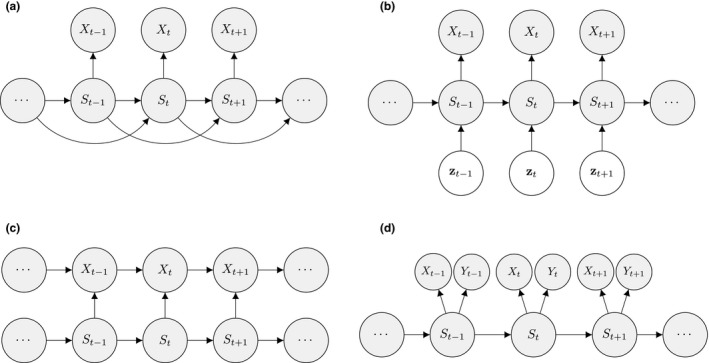
Graphical models associated with different extensions of the basic HMM formulation: (a) state sequence with memory order 2; (b) influence of covariate vectors $z_{1},\ldots ,z_{T}$ on state dynamics; (c) observations depending on both states and previous observations; (d) bivariate observation sequence, conditionally independent given the states.

The dependence assumptions made within the basic HMM are mathematically convenient, but not always appropriate (see Box 2). The Markov property implies that the amount of time spent in a state before switching to another state – the so‐called *sojourn time* – follows a geometric distribution. The most likely length of any given sojourn time hence is one unit, which may not be realistic for certain state processes. The obvious extension is to allow for kth‐order dependencies in the state process (Fig. 4a), such that the state at time t depends on the states at times t‐1,t‐2,…,t‐k. An alternative assumes the state process is ‘semi‐Markov’ with the sojourn time flexibly modelled using any distribution on the positive integers (Choquet *et al*., 2011; van de Kerk *et al*., 2015; King and Langrock, 2016).

HMMs are often used to infer the drivers of ecological state processes by relating the state transition probabilities to explanatory covariates (Fig. 4b). Indeed, any of the parameters of a basic HMM can be modelled as a function of covariates (e.g. sex, age, habitat type, chlorophyll‐a) using an appropriate link function (McCullagh and Nelder, 1989). Link functions $\left(l\right)$ can relate the natural scale parameters $\left(\theta \right)$ to a T×r design matrix of covariates $\left(Z\right)$ and r‐vector of working scale parameters $\left(\beta \in R^{r}\right)$ such that $l\left(\theta \right)=Z\beta$ (see White and Burnham, 1999; MacKenzie *et al*., 2002; Patterson *et al*., 2009, for common examples of link functions in HMMs). When simultaneously analysing multiple observation sequences, heterogeneity across the different sequences can be modelled through explanatory covariates or mixed HMMs that include random effects (Altman, 2007; Schliehe‐Diecks *et al*., 2012; Towner *et al*., 2016).

At the level of the observation process, it is relatively straightforward to relax the conditional independence assumption. For example, it can be assumed that the observation at time t depends not only on the state at time t but also the observation at time t‐1 (Fig. 4c; Langrock *et al*., 2014b; Lawler *et al*., 2019). It is also straightforward to model multivariate observation sequences using multivariate state‐dependent distributions (Choquet *et al*., 2013; Phillips *et al*., 2015; van Beest *et al*., 2019), where it is often assumed that the different variables observed are conditionally independent and a univariate distribution is specified for each of the variables (Fig. 4d). Owing to the Markov property, this does not imply that the individual components are serially independent or mutually independent (Zucchini *et al*., 2016, Chapter 9). However, this assumption is not required and will not always be appropriate, in which case a multivariate distribution should be considered.

## ECOLOGICAL APPLICATIONS OF HIDDEN MARKOV MODELS

In their classic textbook, Begon *et al*. (2006) present the evolutionary foundation of ecology and its superstructure built from individual organisms to populations, communities and ecosystems. At each level of this hierarchy, we will illustrate how HMMs can be used for identifying patterns and dynamics of many different types of ecological state variables that would otherwise be difficult or impossible to observe directly. For each application, we emphasise the two principal components of any HMM – the observation process and the state process – as a conceptual template for ecologists to formulate HMMs in terms of their particular systems of interest.

The observation process in ecological studies is often driven by many factors, including the system state variable(s) of interest, the biotic and/or abiotic components of the system, and study design (Fig. 1). Among the most common types of observation processes in ecology are capture–recapture (Williams *et al*., 2002), DNA sampling (Bohmann *et al*., 2014; Rowe *et al*., 2017; Bálint *et al*., 2018), animal‐borne telemetry (Cooke *et al*., 2004; White and Garrott, 1990; Hooten *et al*., 2017), count surveys (Buckland *et al*., 2004; Charmantier *et al*., 2006; Nichols *et al*., 2009), presence–absence surveys (Koleff *et al*., 2003; MacKenzie *et al*., 2018) and abiotic measurement (e.g. temperature, precipitation, sediment type). These observation processes are not mutually exclusive, can contribute information at different levels of the hierarchy and can be pooled for inference (Schaub and Abadi, 2011; Gimenez *et al*., 2012; Evans *et al*., 2016).

Using Fig. 1 as our expositional roadmap, we begin with applications for individual‐level state dynamics. We then work our way up to the population, community and ecosystem levels. Within each level of the ecological hierarchy, we find it convenient to distinguish ‘existential’, ‘developmental’ and ‘spatial’ states. Although there is inevitably some degree of overlap, particularly at the higher levels of the hierarchy that are inherently spatial, we use this distinction in an attempt to separate states of being that in isolation can be viewed as essentially non‐spatial from state dynamics that are more strictly spatial in nature. We further delineate the non‐spatial states as ‘existential’ based on a fundamental measure of existence at each level of the hierarchy and ‘developmental’ based on state characteristics that can drive the dynamics of this fundamental measure of existence. We employ these categories simply for ease of exposition and view them as neither exhaustive nor mutually exclusive.

Although typically not referred to as HMMs in the ecological literature, several subfields of ecology have been using HMMs for individual‐ to community‐level inference for decades. HMMs have also become standard in biological sequence analysis and molecular ecology (Durbin *et al*., 1998; Barbu and Limnios, 2009; Yoon, 2009), and there is much crossover potential for state‐of‐the‐art bioinformatic methods to other applications in ecology (Jones *et al*., 2006; Tucker and Duplisea, 2012). HMMs are also used for very specialised tasks of relevance to ecology, such as counting annual layers in ice cores (Winstrup *et al*., 2012) or characterising plant architectures (Durand *et al*., 2005). There are therefore many example HMM applications within some areas of ecology, of which only a handful can be covered in the material that follows. However, in other areas the promise of HMMs has only just begun to be recognised.

### Individual level

#### Existential state

At the level of an individual organism, a fundamental measure of existence is to be alive or not (i.e. dead or unborn). We will therefore begin by demonstrating that one of the oldest and most popular inferential tools in wildlife ecology, the Cormack‐Jolly‐Seber (CJS) model of survival (Williams *et al*., 2002), is a special case of an HMM. The CJS model estimates survival probabilities (ϕ) from capture–recapture data. Capture–recapture data consist of n sequences of encounter histories for marked individuals collected through time, where for each individual the observed data are represented as a binary series of ones and zeros. For the CJS model, $X_{t}=1$ indicates a marked individual was alive and detected at time t, while $X_{t}=0$ indicates non‐detection. Marked individuals can either be alive or dead at time t, but the ‘alive’ state is only partially observable and the ‘dead’ state is completely unobservable. Under this observation process, if $X_{t}=1$ it is known that the individual survived from time t‐1 to time t (with probability ϕ) and was detected with probability p. However, when $X_{t}=0$ there are two possibilities: (1) the individual survived to time t (with probability ϕ) but was not detected (with probability 1‐p); or (2) the individual did not survive from time t‐1 to time t (with probability 1‐ϕ).

Although not originally described as such, the CJS model is simply a two‐state HMM that conditions on first capture. Framing the observed and hidden processes within the dependence structure of a basic HMM (Fig. 2), we could for example have:

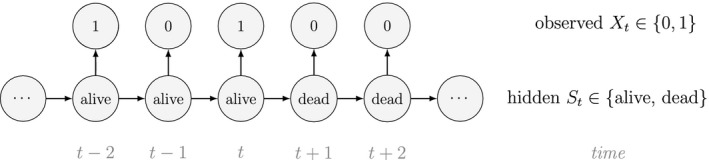

The state‐dependent observation distribution for $X_{t}$ is a simple Bernoulli (i.e. a coin flip) with success probability p if alive and success probability 0 if dead:

$$f\left(X_{t}=x_{t}|S_{t}=i\right)=\left\{\begin{array}{cc} p^{x_{t}}{\left(1‐p\right)}^{1‐x_{t}} & \text{if}\;i=\text{alive}\\ 0^{x_{t}}{\left(1‐0\right)}^{1‐x_{t}}=1‐x_{t} & \text{if}\;i=\text{dead} \end{array}\right)$$

We thus have the initial distribution

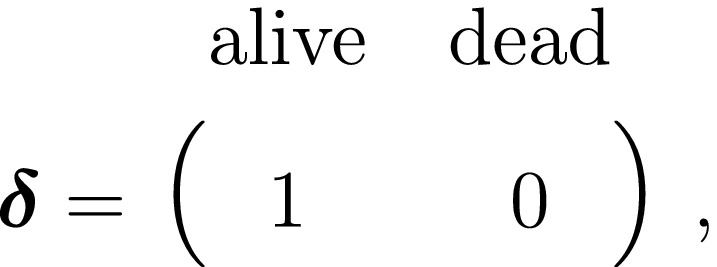

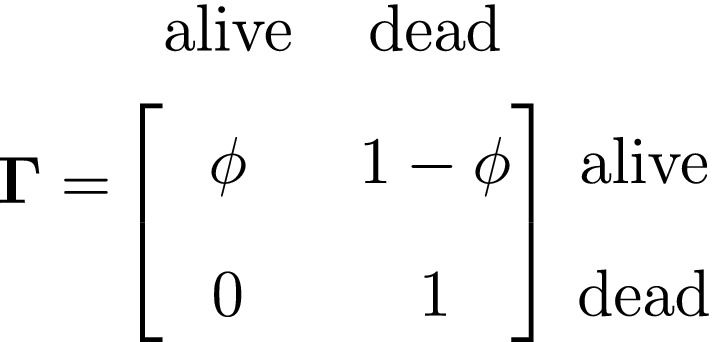

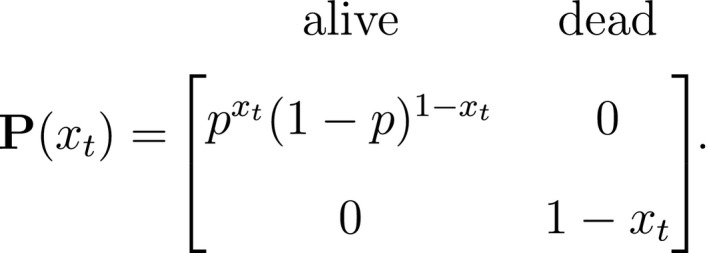

state transition probability matrix and state‐dependent observation distribution matrix

The CJS model is thus a very simple HMM with an absorbing ‘dead’ state and only two unknown parameters (ϕ and p). As an HMM, it can not only be used to estimate survival, but also the point in time when any given individual was most likely to have died (based on local or global state decoding; see Table 1).

The classic Jolly‐Seber capture–recapture model and its various extensions (Pradel, 1996; Williams *et al*., 2002) go a step further by incorporating both birth and death processes. It simply involves extending the two‐state model to an additional ‘unborn’ (UB) state. We could for example now have:

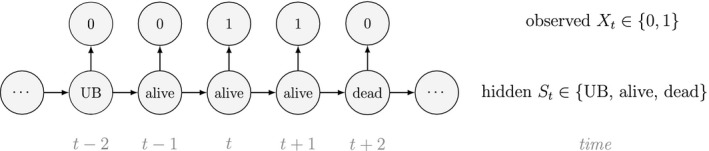

To formulate a three‐state HMM with an additional ‘unborn’ state, we must extend our components for the hidden and observed processes accordingly:

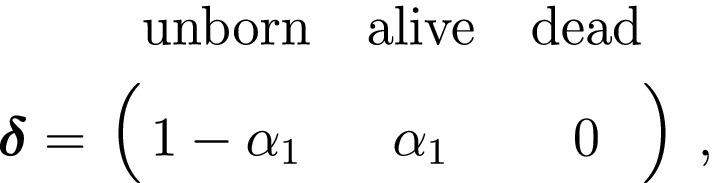

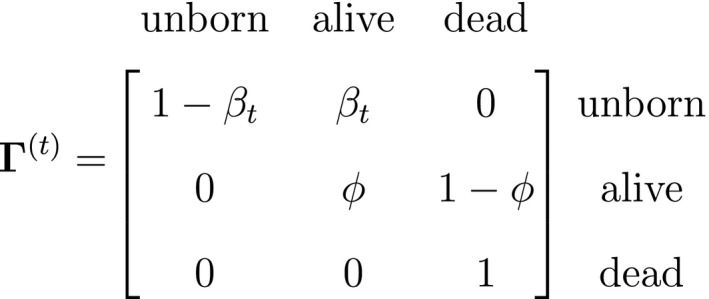

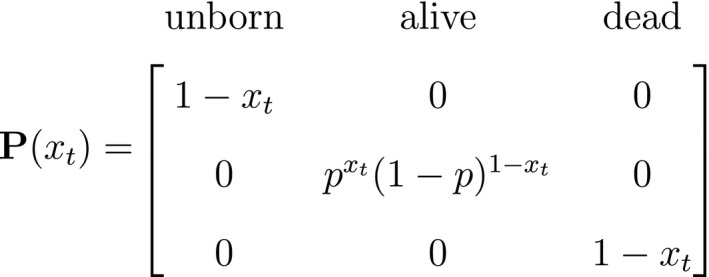

and where

$$\beta_{t}=\left\{\begin{array}{cc} \alpha_{1} & \text{if}\;t=1\\ \frac{\alpha_{t}}{\prod_{l=1}^{t‐1}(1‐\beta_{l})} & \text{if}\;t>1 \end{array}\right),$$

$\alpha_{1}$ is the probability that an individual was already in the population at the beginning of the study, $\alpha_{t}$ is the probability that any given individual was born at time $t\in \left\{2,\ldots ,T\right\}$, and $\beta_{t}$ is the probability that an individual entered the population on occasion t given it had not already entered up to that time. Importantly, note that the two‐state and three‐state HMMs rely on the exact same binary data $\left(X_{t}\in \left\{0,1\right\}\right)$, but we are able to make additional inferences in the three‐state model by re‐formulating the observed and hidden processes in terms of both birth and death. While we have employed these well‐known individual‐level capture–recapture models to initially demonstrate the key idea of linking observed state‐dependent processes to the underlying state dynamics via HMMs, these types of inferences are not limited to traditional capture–recapture observation processes. For example, telemetry and count data can also be used in HMMs describing individual‐level birth and death processes (Schmidt *et al*., 2015; Cowen *et al*., 2017).

#### Developmental state

Individual‐level data often contain additional information about developmental states such as those related to size (Nichols *et al*., 1992), reproduction (Nichols *et al*., 1994), social groups (Marescot *et al*., 2018) or disease (Benhaiem *et al*., 2018). However, assigning individuals to states can be difficult when traits such as breeding (Kendall *et al*., 2012), infection (Chambert *et al*., 2012), sex (Pradel *et al*., 2008) or even species (Runge *et al*., 2007) are ascertained through observations in the field. This difficulty has motivated models for individual histories that can not only account for multiple developmental states (Lebreton *et al*., 2009), but also uncertainty arising from partially or completely unobservable states (Pradel, 2005). Such multi‐state models can be used for testing a broad range of formal biological hypotheses, including host–pathogen dynamics in disease ecology (Lachish *et al*., 2011), reproductive costs in evolutionary ecology (Garnier *et al*., 2016) and social dominance in behavioural ecology (Dupont *et al*., 2015). For example, it is straightforward to extend the capture–recapture HMM to multiple ‘alive’ states parameterised in terms of state‐specific survival probabilities $\left(\phi \right)$ and transition probabilities between these ‘alive’ states $\left(\psi \right)$. Consider a three‐state HMM for capture–recapture data that incorporates reproductive status, where $S_{t}=B$ indicates ‘alive and breeding’ and $S_{t}=\text{NB}$ indicates ‘alive and non‐breeding’:

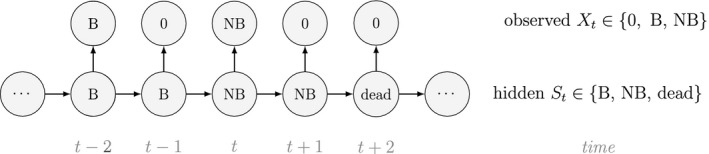

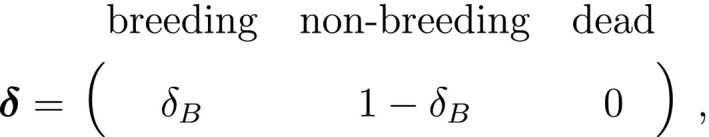

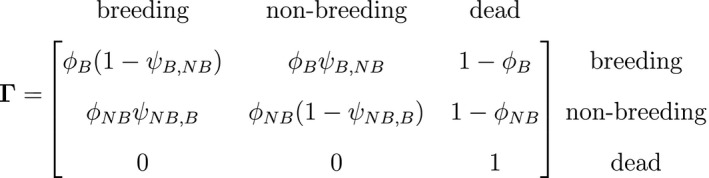

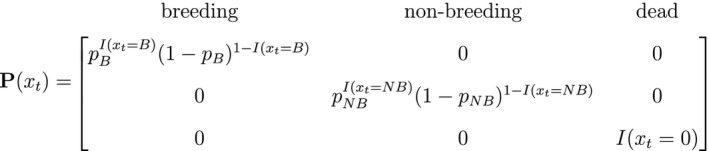

and where $I\left(x_{t}=k\right)$ is an indicator function taking the value 1 when $x_{t}=k$ and 0 otherwise. To assess the costs of reproduction, a biologist will be interested in the probability of breeding in year t, given breeding $\left(\psi_{B,B}=1‐\psi_{B,NB}\right)$ or not $\left(\psi_{NB,B}\right)$ in year t‐1, as well as assessing any differences in survival probability between breeders $\left(\phi_{B}\right)$ and non‐breeders $\left(\phi_{\mathrm{NB}}\right)$. By simply re‐expressing the δ, Γ and $P\left(x_{t}\right)$ components in terms of the specific state and observation processes of interest, such models can be used to infer the dynamics of conjunctivitis in house finches (Conn and Cooch, 2009), senescence in deer (Choquet *et al*., 2011), reproduction in Florida manatees (Kendall *et al*., 2012), interspecific competition between ungulates (Gamelon *et al*., 2020) and life‐history trade‐offs in elephant seals (Lloyd *et al*., 2020). Similar HMMs can also be used to investigate relationships between life‐history traits and demographic parameters that are important in determining the fitness of phenotypes or genotypes (Stoelting *et al*., 2015). Several measures of individual fitness have been proposed, but one commonly used for field studies is lifetime reproductive success (Rouan *et al*., 2009; Gimenez and Gaillard, 2018). These approaches can be readily adapted to quantify other measures of fitness (McGraw and Caswell, 1996; Link *et al*., 2002; Coulson *et al*., 2006; Marescot *et al*., 2018).

Inferences about developmental states are of course not limited to traditional capture–recapture data, and significant advancements in animal‐borne biotelemetry technology have brought many new and exciting opportunities (Cooke *et al*., 2004; Hooten *et al*., 2017; Patterson *et al*., 2017). For example, telemetry location data can be used to identify migratory phases (Weng *et al*., 2007), predation events (Franke *et al*., 2006) or the torpor‐arousal cycle of hibernation (Hope and Jones, 2012). The multi‐state (i.e. hidden Markov) movement model is often used to infer these types of movement behaviour modes from trajectories in two‐dimensional space, where the observations are typically expressed in terms of the bivariate sequence of Euclidean distances (or ‘step lengths’) and turning angles between consecutive locations (Franke *et al*., 2004; Morales *et al*., 2004). For a model involving N=2 states that assumes conditional independence between step length ($X_{t}$; in meters) and turning angle ($Y_{t}$; in radians) as in Fig. 4d, we could for example have:

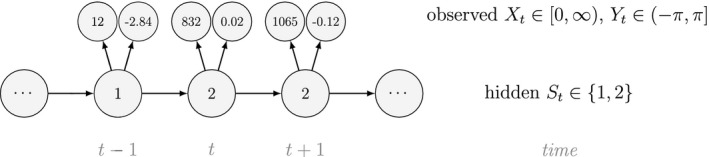

These states could correspond to ‘resident’ (state 1) and ‘transient’ (state 2) behavioural phases, such that within state 2 the movements tend to be longer and directionally persistent (i.e. with turning angles concentrated near zero). When assuming conditional independence of the observations, the bivariate state‐dependent distribution for $\left(X_{t},Y_{t}\right)$ is simply the product of two univariate state‐dependent distributions,

$$f\left(x_{t},y_{t}|S_{t}=i\right)=f\left(x_{t}|S_{t}=i\right)f\left(y_{t}|S_{t}=i\right).$$

These univariate distributions are typically assumed to be the gamma or Weibull distribution for step length and the von Mises or wrapped Cauchy distribution for turning angle. Unlike our previous examples so far, the number of underlying states in these types of HMMs is generally not clear *a priori* and needs to be selected based on both biological and statistical criteria (Pohle *et al*., 2017). Another difference is that there is often no predetermined structure in the state transition probability matrix,

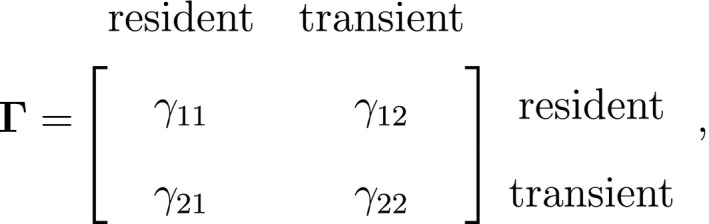

and all entries are freely estimated (but still subject to $\sum_{j=1}^{N}\gamma_{ij}=1$). As a consequence, the characteristics of the model states as represented by the state‐dependent distributions are fully data driven, and hence may not correspond exactly to biologically meaningful entities (see IMPLEMENTATION, CHALLENGES AND PITFALLS).

**Figure 5:**
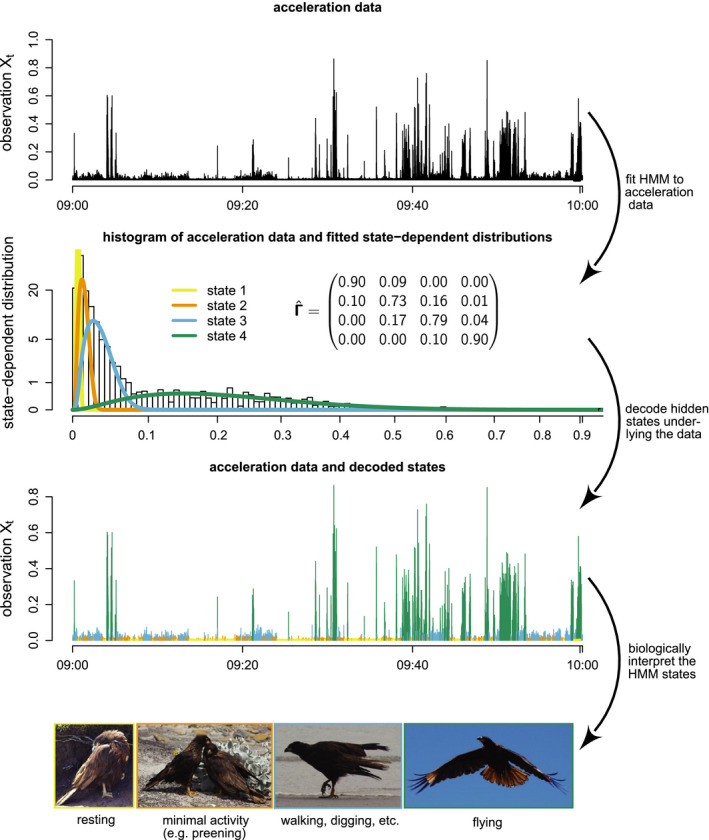
Illustration of a possible workflow when using an HMM to infer behavioural modes from the vector of dynamic body acceleration data of a striated caracara (*Phalcoboenus australis*) over a period of 60 min (see Fahlbusch & Harrington, 2019, for data details). Four behavioural modes were identified and biologically interpreted to be associated with resting (yellow), minimal activity (orange), moderate activity (blue) and flying (green).

Similar HMMs for animal movement have been used, *inter alia*, to identify wolf kill‐sites (Franke *et al*., 2006), the relationship between southern bluefin tuna behaviour and ocean temperature (Patterson *et al*., 2009), activity budgets for harbour seals (McClintock *et al*., 2013), hunting strategies of white sharks (Towner *et al*., 2016), the behavioural response of northern gannets to frontal activity (Grecian *et al*., 2018) and how common noctules adjust their space use to the lunar cycle (Roeleke *et al*., 2018). Driven by the influx of new biotelemetry sensor technology, HMMs have also been used to analyse the sequences of dives of marine animals (Hart *et al*., 2010; Quick *et al*., 2017; DeRuiter *et al*., 2017; van Beest *et al*., 2019). The remote collection of activity data at potentially very high temporal resolutions using accelerometers is another emerging application area (Diosdado *et al*., 2015; Leos‐Barajas *et al*., 2017b; Papastamatiou *et al*., 2018a,b; Adam *et al*., 2019b). These HMM formulations are conceptually very similar to the movement model outlined above, with the state process corresponding to behavioural modes (or at least proxies thereof), and the activity data represented by the state‐dependent process. Fig. 5 illustrates a possible workflow for inferring four behavioural modes from high‐resolution accelerometer data collected from a striated caracara (*Phalcoboenus australis*) over a period of 1 hour. Here the vector of dynamic body acceleration was used as a univariate summary of the three‐dimensional raw acceleration data, and a gamma distribution was used for the state‐dependent observation process. In this example, the HMM can be regarded as a clustering scheme which maps observed input data to unobserved underlying classes with biological interpretations roughly corresponding to ‘resting’, ‘minimal activity’ (e.g. preening), ‘moderate activity’ (e.g. walking, digging) and ‘flying’. Complete details of this analysis, including each step of the workflow and example R (R Core Team, 2019) code, can be found in the Supplementary Tutorial.

#### Spatial state

HMMs can also be used for inferences about the unobserved spatial location of an individual. For example, capture–recapture data can consist of sequences of observations arising from a set of discrete spatial states, where these often refer to ecologically important geographic areas, such as wintering and breeding sites for migratory birds (Brownie *et al*., 1993) or spawning sites for fish (Schwarz *et al*., 1993). For a three‐state HMM with two sites (A and B), where $S_{t}=A$ indicates ‘alive at site A’ and $S_{t}=B$ indicates ‘alive at site B’, we could for example have:

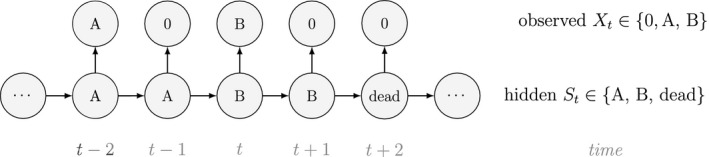

Clearly, this discrete‐space HMM is structurally identical to the multi‐state capture–recapture HMMs already described in the previous section; the only difference is the state transition probability parameters are now interpreted as site‐specific survival and movement probabilities between the sites (e.g. fidelity or dispersal; Lagrange *et al*., 2014; Cayuela et al., 2020). Based on global state decoding, these HMMs can therefore also be used to infer the most likely spatial state for periods when an individual was alive but its location was not observed.

Another important application of HMMs is for geolocation based on indirect measurements that vary with space, such as light, pressure, temperature and tidal patterns (Thygesen *et al*., 2009; Rakhimberdiev *et al*., 2015). Although too technical to be described in detail here, geolocation HMMs can be particularly useful for inferring individual location from archival tag data (Basson *et al*., 2016). These HMMs have even been extended to include state‐switching behaviours such as those described in the previous section (Pedersen *et al*., 2008, 2011b). Animal movement behaviour HMMs have also been extended to accommodate partially observed location data common to marine mammal satellite telemetry studies (Jonsen *et al*., 2005; McClintock *et al*., 2012).

### Population level

We consider two ways that inference on the population level can arise: (1) an individual‐level model, based on data from multiple individuals (e.g. capture–recapture), quantitatively connected to a population‐level concept through an explicit model; or (2) a population‐level model, based on population‐level data (e.g. counts or presence–absence), with no explicit model for processes at the individual level.

#### Existential state

A fundamental existential state at the population level is abundance, the number of individuals alive in a population at a particular point in time. A common way to infer this using capture–recapture HMMs is to formally link abundance to the individual‐level processes (e.g. survival, recruitment) that drive its dynamics. Intuitively, the abundance model specifies how many individuals go through the life history specified by the HMM. For the abundance component, the key pieces of information are the number of individuals in the population that were detected at least once $\left(n\right)$ and the probability of being detected at least once, given an individual was alive at any time during the study $\left(p^{*}\right)$. The former is observed while the latter can be calculated as

$$p^{*}=1‐\delta P\left(x_{1}=0\right)\Gamma^{\left(1\right)}P\left(x_{2}=0\right)\Gamma^{\left(2\right)}\cdots \Gamma^{\left(T‐1\right)}P\left(x_{T}=0\right)1$$

using notation for the Jolly‐Seber HMM presented in Individual level. This HMM formulation is equivalent to the original Jolly‐Seber open population model (shown in Glennie *et al*., 2019), where population abundance at each time t is derived from the individual‐level process parameters.

Instead of inducing changes in abundance through individual‐level HMMs, abundance itself can be modelled as the hidden state within an HMM (Schmidt *et al*., 2015; Cowen *et al*., 2017; Besbeas and Morgan, 2019). Here population dynamics are inferred from population‐level surveys (Buckland *et al*., 2004), where the observation process can include counts or other quantities that are noisy measurements of the true abundance (the hidden state), and the state transition probability matrix $\left(\Gamma \right)$ is naturally formulated in terms of the well‐known Leslie matrix for population growth (Caswell, 2001). For example, for imperfect count data $X_{t}\in \left\{0,1,2,\ldots \right\}$ that were collected from a population of true size $S_{t}\in \left\{0,1,\ldots ,N_{\max}\right\}$ (note the requirement to specify a maximum possible population size $N_{\max}$), we could have:

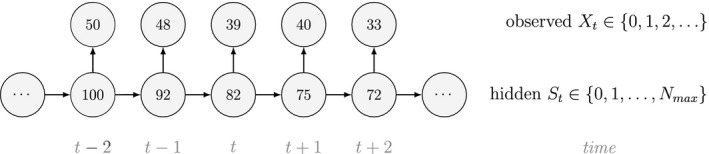

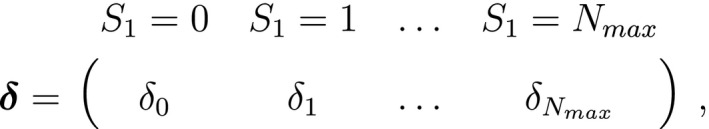

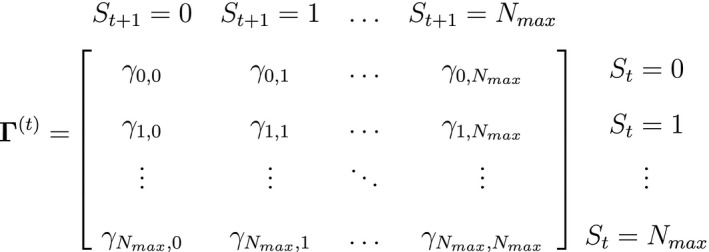

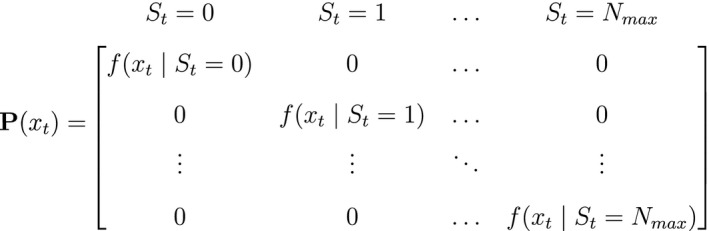

and

Each state transition probability $\left(\gamma_{\mathrm{ij}}\right)$ describes the population dynamics from time t to time t+1 and can be parameterised in terms of survival, reproduction, emigration, the current population size $\left(S_{t}\right)$ and any additional population structure (e.g. sex or age classes; see Population level ‐ Developmental state). The state‐dependent distributions $f(x_{t}|S_{t}=i)$ can take many different forms depending on the specific observation process, but common choices for count data are binomial or Poisson models (Schmidt *et al*., 2015; Besbeas and Morgan, 2019). Sometimes count data alone can be insufficient for describing complex population processes, and this has led to integrated population modelling (Schaub and Abadi, 2011) that uses auxiliary data such as capture–recapture, telemetry or productivity data (Schmidt *et al*., 2015; Besbeas and Morgan, 2019).

#### Developmental state

Populations have more structure than simply their overall abundance or density. Sex, age demographics, size of breeding sub‐population, fitness of individuals, and behavioural or genetic heterogeneity all have an impact on the development of a population (Seber and Schofield, 2019). Many of these processes can be accounted for within the HMM framework presented in the previous section for individual‐level data. As before, the idea is to extend the ‘alive’ state to a more complex network of states whose state‐dependent distributions and transitions match the structure in the population. Combinations of these individual attributes provide the opportunity to build a rich state process to describe the population dynamics. This framework is built around the idea that individuals are the singular units that together drive population change, but there has also been increasing use of HMMs from a different viewpoint: that of evolutionary processes at lower levels of organisation (e.g. genes).

With recent advances in genetic sequencing, the need for interpreting and modelling biological sequences (e.g. protein or DNA) has boosted the development of HMMs in molecular ecology (Durbin *et al*., 1998; Boitard *et al*., 2009; Yoon, 2009; Ghosh *et al*., 2012). Many of these applications use HMMs strictly as a tool for biological sequence analysis (e.g. identifying species from DNA barcodes; Hebert *et al*., 2016) and are too technical to delve into detail here, but HMMs for molecular sequence data are commonly formulated in terms of evolutionary state dynamics, including for example speciation and extinction (Hobolth *et al*., 2007; Soria‐Carrasco *et al*., 2014; Crampton *et al*., 2018; Olajos *et al*., 2018), hybridisation (Schumer *et al*., 2018; Palkopoulou *et al*., 2018), mutualism (Werner *et al*., 2018), hidden drivers of diversification (Caetano *et al*., 2018) and evolutionary rates among sites (Felsenstein and Churchill, 1996).

Telemetry locations are another form of individual‐level data that, when combined across individuals, can provide population‐level inferences about movement, space use and resource selection (Hooten *et al*., 2017). As such, telemetry data can be well suited for addressing hypotheses related to intraspecific interactions. While such applications are still relatively rare, location data have been used in HMMs investigating intraspecific competition in marine mammals (Breed *et al*., 2013), herding in ungulates (Langrock *et al*., 2014a) and social behaviour in fish (Bode and Seitz, 2018).

Similar to approaches for inferring population‐level developmental states from individual‐level data, a rich structure can also be specified within an HMM for population‐level data. Multiple states and processes can be represented: age classes/survival, size classes/growth, sex/birth, genotypes and metapopulations are all states or networks of states with specified connections (Newman *et al*., 2014). Such HMMs can be informed by a wide variety of population‐level observations, for example counts of plants (Borgy *et al*., 2015) or animals (Schmidt *et al*., 2015), as well as auxiliary individual‐level observations (Besbeas and Morgan, 2019). From this general viewpoint, HMMs can be seen as the structure behind open population N‐mixture models (Schmidt *et al*., 2015; Cowen *et al*., 2017), distance sampling models (Sollmann *et al*., 2015) and approximate state‐space population dynamics models (Besbeas and Morgan, 2019).

#### Spatial state

The spatial state of a population can be conceived as a surface (or map) quantifying density at each point in space, and population models for individual‐level data can be extended to allow density to change over space (Borchers and Efford, 2008). Inferring density as a spatial population state, however, requires spatial information within the data. Spatial capture–recapture surveys (Royle *et al*., 2013), an extension of capture–recapture, collect precisely these data. Spatial capture–recapture HMMs can be formulated in terms of survival, recruitment, movement and population density (Royle *et al*., 2018; Glennie *et al*., 2019) and are readily extendable for relating environment and population distribution across space, including how distribution is affected by landscape connectivity, dispersal, resource selection or environmental impacts such as oil spills (McDonald *et al*., 2017; Royle *et al*., 2018).

A different viewpoint is to consider population‐level data that are commonly collected over both space and time: presence–absence data. These data provide information on a population's spatial state that is not derived from abundance and arise from the monitoring of spatial units for the (apparent) presence or absence of a species. One of the most popular tools for analysing these data are patch (or site) occupancy models, which can be used to infer patterns and dynamics of species occurrence while accounting for imperfect detection (MacKenzie *et al*., 2018). As with capture–recapture models, patch occupancy models are also HMMs (Royle and Kéry, 2007; Gimenez *et al*., 2014) where, instead of the state dynamics of individual organisms, the hidden process describes the state dynamics of sites. Let $S_{t}=O$ indicate ‘occupied’ and $S_{t}=U$ indicate ‘unoccupied’, where the species can be detected $\left(X_{t,k}=1\right)$ or not $\left(X_{t,k}=0\right)$ during multiple visits k=1,…,K to each site, with the following representation:

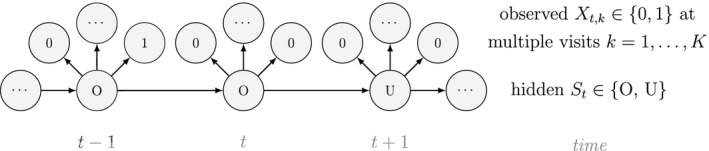

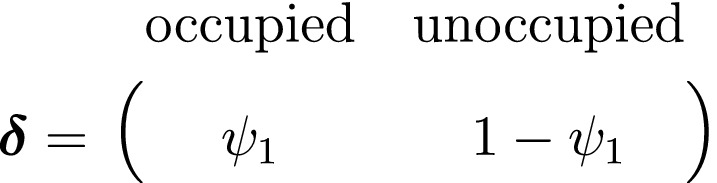

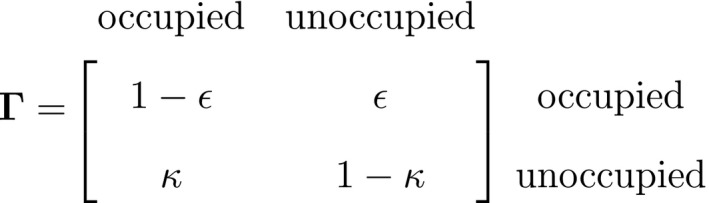

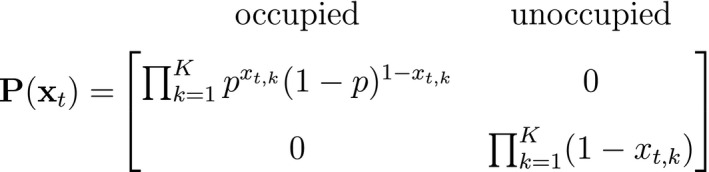

and where $\psi_{1}$ is the initial patch occupancy probability at time t=1, p is the species detection probability at each occupied patch and Γ is composed of the local colonisation $\left(\kappa \right)$ and extinction $\left(\epsilon \right)$ probabilities. Single‐season (or static) occupancy models (MacKenzie *et al*., 2002) are obtained as a special case with T=1 or ϵ=κ=0 (Gimenez *et al*., 2014). This HMM can not only be used to estimate patch occupancy, extinction and colonisation probabilities, but also the most likely state and times of any colonisation or extinction events within a patch. The flexibility of the HMM formulation allows patch occupancy to be conveniently extended to cope with site‐level heterogeneity in detection using finite mixtures (Louvrier *et al*., 2018) or a discrete measure of population density (Gimenez *et al*., 2014; Veran *et al*., 2015) and even false positives due to species misidentification (Miller *et al*., 2011; Louvrier *et al*., 2019). Just as with multi‐state capture–recapture HMMs (see Population level ‐ Developmental state), species occurrence HMMs can be readily extended to multiple ‘occupied’ states accommodating reproduction (MacKenzie *et al*., 2009; Martin *et al*., 2009), disease (McClintock *et al*., 2010) and other (meta‐)population dynamics (Lamy *et al*., 2013).

Inferences from HMMs for presence–absence data are not limited to occupancy models that account for imperfect species detection. For example, Pluntz *et al.,* (2018) developed an HMM characterising seed dormancy, colonisation and germination in annual plant metapopulations based entirely on presence–absence observations of standing flora. In their study, the presence of a completely unobservable soil seed bank was the hidden state of interest, and they modified the dependence structure of a basic HMM such that the seed bank state dynamics at time t depended not only on the seed bank state at time t‐1, but also on the presence or absence of standing flora at time t. Let $S_{t}=\text{AA}$ indicate ‘seed bank absent at time t‐1, flora absent at time t’, $S_{t}=\text{PA}$ indicate ‘seed bank present at time t‐1, flora absent at time t’ and $S_{t}=\text{PP}$ indicate ‘seed bank present at time t‐1, flora present at time t’, where standing flora is present $\left(X_{t}=1\right)$ or not $\left(X_{t}=0\right)$ during visit t to each site and is assumed to be detected without error. We could for example have:

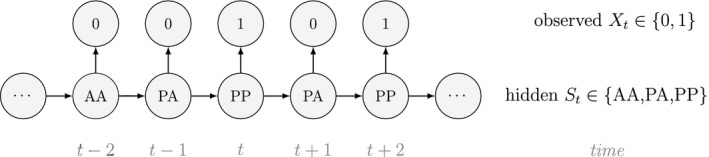

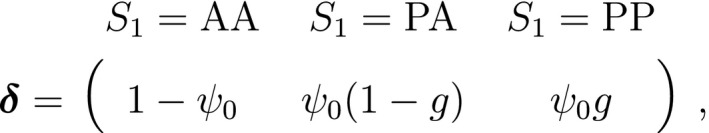

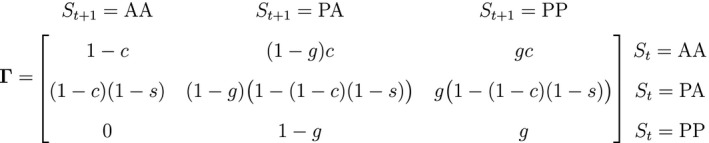

where $\psi_{0}$ is the probability that a seed bank was present the year before the first observation, g is the probability of germination and survival to reproduction, s is the probability of seed bank survival, c is the probability of external colonisation and $P\left(x_{t}\right)$ is a 3×3 diagonal matrix of ones. Similar formulations could be applied to other organisms with dormant life stages (e.g. fungi, crustaceans).

### Community level

Community‐level studies often focus on a subset of species based on taxonomy, trophic position or particular interactions of interest, and the diversity of topics addressed in community ecology reflects its large scope (Vellend, 2010, 2016). Here we will only scratch the surface of two study systems that can be formulated as HMMs for multi‐species presence–absence data commonly collected from field surveys or (e)DNA samples: (1) patch systems composed of (potentially) many species; and (2) patch systems composed of a few (possibly interacting) species.

#### Existential state

A fundamental measure of biodiversity is the number of species within a community (species richness). This community‐level state is often unobservable in studies of natural systems (Dorazio *et al*., 2006), even for communities composed entirely of sessile organisms (Conway‐Cranos and Doak, 2011; Chen *et al*., 2013). Multi‐species occupancy HMMs expand single‐species occupancy HMMs (see Population level) to the community level using presence–absence data for each species that could (potentially) occupy the sampling units within a study area (MacKenzie *et al*., 2018, Chapter 15). By combining single‐species HMMs, either independently or by sharing common parameters among species (Evans *et al*., 2016; Guillera‐Arroita, 2017), community‐level attributes (e.g. species richness) and species‐level attributes (e.g. patch occupancy) can be integrated within a single modelling framework (Royle and Dorazio, 2008, Chapter 12). By jointly modelling species‐ and community‐level processes, the approach proposed by Dorazio and Royle (2005) and its extensions (reviewed by Kery and Royle, 2015, Chapter 11) facilitate the simultaneous testing of formal hypotheses about factors influencing occupancy (Rich *et al*., 2016; Tenan *et al*., 2017), species richness (Sutherland *et al*., 2016) and their dynamics through time (Russell *et al*., 2009; Dorazio *et al*., 2010), with important consequences for conservation and management (Zipkin *et al*., 2010). Although these community dynamics models are typically fitted using hierarchical Bayesian methods and not explicitly referred to as HMMs, they share the same properties and can be similarly decomposed in terms of δ, Γ and $P\left(x_{t}\right)$. Viewing the species richness of a community as analogous to the abundance of a population, HMM formulations similar in spirit to those described in Population level could account for species that were never detected (*sensu* Dorazio *et al*., 2006).

#### Developmental state

Many community‐level attributes can be constructed from ‘metacommunity’ HMMs for species richness at both the community and metacommunity level (Dorazio and Royle, 2005; Kery and Royle, 2015, Chapter 11). Species richness at each site is the α diversity metric, and total richness in the whole metacommunity is the γ diversity (Magurran, 2004, Chapter 6). A possible metric for the β diversity is the similarity Jaccard index: the proportion of species that occur at two sites among the species that occur at either site. Multi‐species occupancy models have also been used to address variation in community attributes within distinct regions using Hill numbers for species richness, Shannon diversity and Simpson diversity (Broms *et al*., 2015; Sutherland *et al*., 2016; Tenan *et al*., 2017; Boron *et al*., 2019). Dynamic multi‐species occupancy HMMs can provide inferences about changes in community composition and structure over time, entry (or ‘turnover’) probabilities of ‘new’ species into the community and species ‘extinction’ probabilities from the community (Russell *et al*., 2009; Dorazio *et al*., 2010). Although to our knowledge this has not yet been attempted, community assembly or succession dynamics could naturally be parameterised in terms of such quantities within a multi‐state, multi‐species HMM describing transitions among different community states (e.g. disturbed, climax). Community structure and composition also depend on interspecific interactions, and multi‐species occupancy HMMs can empirically test for any such evidence (Gimenez *et al*., 2014; Rota *et al*., 2016; Davis *et al*., 2018; MacKenzie *et al*., 2018; Marescot *et al*., 2020). To date these co‐occurrence models have mostly been used to infer predator–prey interactions (Miller *et al*., 2018b; Murphy *et al*., 2019). Other emerging frameworks for inferences about processes that structure communities could also potentially be formulated as HMMs to account for observation error in presence–absence or count data (Ovaskainen *et al*., 2017).

#### Spatial state

Understanding geographic variation in the size and structure of communities is one of the major goals in ecology. While we have so far focused on some of the more ‘non‐spatial’ aspects of community‐level inference, all multi‐species presence–absence HMMs are of course inherently spatial and also describe community distribution. Dynamic multi‐species occupancy models provide inferences about changes in community distributions (Russell *et al*., 2009; Dorazio *et al*., 2010), and, when spatio‐temporal interactions between species are of primary interest, dynamic co‐existence HMMs can incorporate local species extinction and colonisation to investigate interspecific drivers of co‐occurrence dynamics and community distribution (Fidino *et al*., 2019; Marescot *et al*., 2020). As a final illustrative example, suppose we have the states $S_{t}=A$ (respectively $S_{t}=B$ and $S_{t}=\text{AB}$) for ‘site occupied by species A’ (respectively by species B and by both species) and $S_{t}=U$ indicates ‘unoccupied site’. Define $X_{t,k}\in \left\{0,1,2,3\right\}$, where 0 indicates neither species was detected, 1 indicates only species A was detected, 2 indicates only species B was detected and 3 indicates both species were detected on the kth visit at time t. We could for example have:

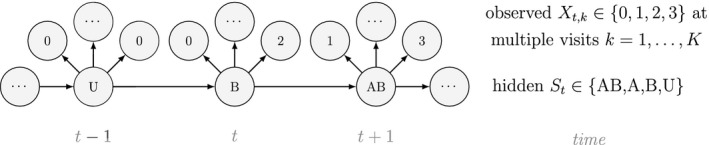

This model is more complex than previous examples, but it can still be readily expressed in terms of δ, Γ and $P\left(x_{t}\right)$ for inferring patterns and drivers of species co‐existence distribution dynamics (see Appendix A in Supplementary Material).

### Ecosystem level

Despite the well‐recognised need for reliable inferences about broad‐scale ecological dynamics in the face of climate change and other challenges (Turner *et al*., 1995), HMMs have thus far seldom been applied at the ecosystem level. This is likely attributable to many factors, including the difficulty of obtaining and integrating observational data at the large spatio‐temporal scales required (Jones *et al*., 2006; Bohmann *et al*., 2014; Dietze *et al*., 2018; Estes *et al*., 2018; Compagnoni *et al*., 2019). Although there are fewer examples in the literature, HMMs have been used to make ecosystem‐level inferences about stability and regime shifts (Gal and Anderson, 2010; Gennaretti *et al*., 2014; Economou and Menary, 2019), climate‐driven community and disease dynamics (Moritz *et al*., 2008; Martinez *et al*., 2016; Miller *et al*., 2018a), the effects of management action on habitat dynamics (Breininger *et al*., 2010), climatic niches (Tingley *et al*., 2009) and ecosystem health (Xiao *et al*., 2019). HMMs are also frequently used by atmospheric scientists, hydrologists and landscape ecologists to describe regional‐ to global‐scale ecosystem processes such as precipitation (Zucchini and Guttorp, 1991; Srikanthan and McMahon, 2001), streamflow (Jackson, 1975; Bracken *et al*., 2014), wetland dynamics (Siachalou *et al*., 2014) and land cover dynamics (Aurdal *et al*., 2005; Lazrak *et al*., 2010; Trier and Salberg, 2011; Abercrombie and Friedl, 2015; Siachalou *et al*., 2015). While many of these examples tend to focus on a few specific biotic and/or abiotic components in which to frame ecosystem state dynamics, we can envision future applications adopting a more holistic approach that integrates increasingly more complex ecosystem‐level processes with observational data arising from a variety of sources and spatio‐temporal scales (see FUTURE DIRECTIONS).

## IMPLEMENTATION, CHALLENGES AND PITFALLS

### Software

Recent advances in computing power and user‐friendly software have made the implementation of HMMs much more feasible for practitioners. However, the features and capabilities of the software are varied, and it can be challenging to determine which software may be most appropriate for a specific objective. We briefly describe some of the HMM software currently available, limiting our treatment to freely available R (R Core Team, 2019) packages and stand‐alone programs that we believe are most accessible to ecologists and non‐statisticians. While most HMM packages in R include data simulation, parameter estimation and state decoding for an arbitrary number of system states, they differ in many key respects (Table 2). Some of the more general packages provide greater flexibility for specifying state‐dependent probability distributions (Visser and Speenkenbrink, 2010; Jackson, 2011; Harte, 2017; McClintock and Michelot, 2018). One of the earliest and most flexible HMM packages, depmixS4 (Visser and Speenkenbrink, 2010), can accommodate multivariate HMMs, multiple observation sequences, parameter covariates, parameter constraints and missing observations. Similar to depmixS4 in terms of features and flexibility, momentuHMM (McClintock and Michelot, 2018) can also be used to implement mixed HMMs (DeRuiter *et al*., 2017), hierarchical HMMs (Leos‐Barajas *et al*., 2017a; Adam *et al*., 2019a), zero‐inflated probability distributions (Martin *et al*., 2005) and partially observed state sequences. In addition to the R packages presented in Table 2, there are numerous R and stand‐alone software packages that are less general and specialise on particular HMM applications in ecology, as well as general statistical programs with which these types of models can be relatively easily implemented (see Appendix B in Supplementary Material).

**Table 2:** Features of HMM packages available in the R environment for statistical computing, including capabilities for multiple observation sequences (‘Multiple sequences’), multivariate HMMs (‘Multivariate’), mixed HMMs (‘Mixed’), hierarchical HMMs (‘Hierarchical’), hidden semi‐Markov models (‘Semi‐Markov’), parameter covariate modelling (‘Covariates’), parameter constraints (‘Constraints’), missing observations (‘Missing data’) and state‐dependent probability distributions

Package	Multiple sequences	Multivariate	Mixed	Hierarchical	Semi‐Markov	Covariates	Constraints	Missing data	Reference
aphid	✓								Wilkinson (2019)
depmixS4	✓	✓				δ,f,γ	δ,f,γ	✓	Visser and Speenkenbrink (2010)
HiddenMarkov						$f^{*}$			Harte (2017)
HMM									Himmelmann (2010)
hsmm					✓				Bulla and Bulla (2013)
LMest	✓	✓	✓			$f^{†}$ or δ,γ		✓	Bartolucci *et al.,* (2017)
mhsmm	✓				✓			✓	O'Connell and Hojsgaard (2011)
momentuHMM	✓	✓	✓	✓		δ,f,γ,π	δ,f,γ,π	✓	McClintock and Michelot (2018)
msm	✓	✓				$f^{‡},\gamma$	f,γ	✓	Jackson (2011)
RcppHMM									Cardenas‐Ovando *et al.,* (2017)
seqHMM	✓	✓	✓			π	δ,γ	✓	Helske and Helske (2019)

	State‐dependent probability distributions																	
	Bernoulli	Beta	Binomial	Categorical	Custom	Exponential	Gamma	Lognormal	Logistic	Negative binomial	Normal	Multivariate normal	Truncated normal	Poisson	Student's t	Von Mises	Weibull	Wrapped Cauchy
aphid				✓														
depmixS4			✓	✓	✓		✓				✓			✓				
HiddenMarkov		✓	✓		✓	✓	✓	✓	✓		✓			✓				
HMM				✓														
hsmm	✓										✓			✓	✓			
LMest				✓							✓	✓						
mhsmm					✓						✓	✓		✓				
momentuHMM	✓	✓		✓		✓	✓	✓	✓	✓	✓	✓		✓	✓	✓	✓	✓
msm	✓	✓	✓	✓		✓	✓	✓		✓	✓		✓	✓	✓		✓	
RcppHMM				✓							✓	✓		✓				
seqHMM				✓														

‘Covariates’ and ‘Constraints’ can pertain to initial distribution $\left(\delta \right)$, state‐dependent probability distribution $\left(f\right)$, state transition probability $\left(\gamma \right)$ and/or mixture probability $\left(\pi \right)$ parameters. Several packages facilitate extensions for user‐specified state‐dependent probability distributions that require no modifications to the package source code (‘custom’).

* Covariates are only permitted on state‐dependent distribution location parameters for the binomial, gamma, normal and Poisson distributions.

^†^ Covariates are only permitted on state‐dependent categorical distribution parameters.

^‡^ Covariates are only permitted on state‐dependent distribution location parameters.

[Corrections added on 10 November 2020, after first online publication: Table 2 has been updated.]

### Challenges and pitfalls

HMMs are natural candidates for conducting inference related to a wide range of ecological phenomena, but they are not a panacea (see Box 2). There are many ecological processes that cannot be faithfully characterised under the simplifying assumptions of HMMs, in which case other latent variable models may be more appropriate (see Box 1). When HMMs are appropriate, it can be challenging to tailor HMMs to real data, even when using user‐friendly software packages. Here we briefly highlight those issues that, based on our experience, constitute the key challenges when using HMMs to analyse ecological data. Other important aspects of statistical practice that are not unique to HMMs, such as model checking and selection (e.g. Zucchini *et al*., 2016, Chapter 6), are covered in more detail in the Supplementary Tutorial.

Depending on the complexity of the state and observation processes, various modelling decisions may need to be made. Among these are the number of states to include, whether to incorporate covariates for the model parameters and whether the basic dependence structure is sufficient. These decisions tend to be case‐dependent and require expert knowledge of the system of interest, so we make no attempt to provide general guidance in this respect. However, in some cases the model structure may be a direct consequence of the ecological process. For example, in the CJS model, the two states (alive or dead) and also the state‐dependent (Bernoulli) distributions follow immediately from the capture–recapture process. In situations with more complex data, such as multivariate time series related to animal behaviour (DeRuiter *et al*., 2017; Ngô *et al*., 2019; van Beest *et al*., 2019), it takes experience and a good intuition both for the data and for the HMM framework to identify an adequate model formulation (Pohle *et al*., 2017).

Unlike other statistical models such as linear regression, there is no analytical solution for HMM parameter estimation. One must therefore resort to numerical procedures, all of which involve technical challenges: local maxima for maximum likelihood estimation (Myung, 2003), or label switching (Jasra *et al*., 2005) and poor mixing (Brooks *et al*., 2011) for MCMC sampling. Any increase in model complexity with respect to the number of states or the parameters tends to rapidly exacerbate these problems. When working with HMMs, it is thus important to develop an appreciation for these challenges and the associated risks. For maximum likelihood estimation, the risk of false convergence to a local rather than the global maximum of the likelihood must not be underestimated. In addition to the general advice to avoid overly complex models (Lavine, 2010; Cole, 2019), the main strategy to reduce this risk is to try many initial parameter vectors within the maximisation.

While it is tempting to interpret the states of an HMM fitted to ecological data as biologically meaningful entities, this is not always justifiable. Outside the standard capture–recapture or species occurrence applications, HMMs are often applied in an unsupervised learning context (see Figs 3 and 5, Supplementary Tutorial), such that the state characteristics are completely data driven rather than pre‐defined (Leos‐Barajas *et al*., 2017b). The model then picks up the *statistically* most relevant modal patterns in the data, and these may or may not correspond closely to *ecologically* meaningful states. It is thus important not to over‐interpret the model states, as in some cases they may only be crude proxies for the ecological system states of interest. A classic example is the simple N=2 state HMM for animal movement behaviour based on step lengths and turning angles (Morales *et al*., 2004), where evidence of an area‐restricted search‐type state is often labelled as ‘foraging’. Although for many animals area‐restricted search is commonly associated with foraging, one usually cannot definitively conclude when an individual was actually foraging based solely on location data. Furthermore, while it can be useful to refer to these modalities using descriptive terms such as ‘foraging’ (or ‘resident’) and ‘searching’ (or ‘transient’), this does not mean that an animal has only two modes of behaviour.

## FUTURE DIRECTIONS

**Figure 6:**
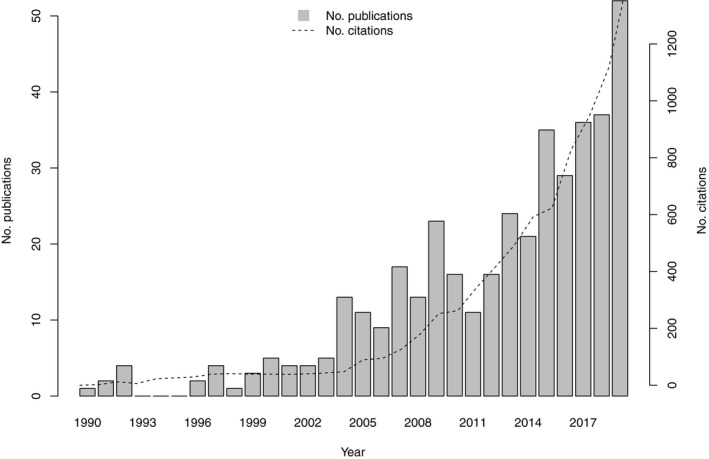
Number of publications (left axis) and total number of times these publications were cited (right axis) per year based on a Web of Science search for ‘hidden Markov’ conducted within the categories of ‘Biology’, ‘Ecology’, ‘Marine Freshwater Biology’ and ‘Zoology’ on 7 July 2020.

We have highlighted many realised and potential applications of HMMs in ecology. We anticipate increased application and development of HMMs as ecologists continue to discover how this relatively simple and flexible class of statistical models can reveal complex state dynamics that are inherently difficult to observe. Indeed, a Web of Science search for ‘hidden Markov’ suggests a rapidly increasing awareness of these models within the ecological community (Fig. 6). Given differences in terminology and a tendency for ecologists to use HMMs without explicitly referring to them as such, the use of HMMs is surely becoming even more widespread.

In order for the power and flexibility of HMMs to be harnessed by the broader ecological community, researchers must first be able to recognise the limitations of their data and how these can be leveraged by formally linking observable phenomena to the actual ecological processes of interest. Such hierarchical modelling exercises are critical to reliable inference (Royle and Dorazio, 2008; Kery and Royle, 2015), and it is no coincidence that HMMs have independently ‘evolved’ in different ecological contexts over the years. By assuming a discrete state space with basic dependence structures, HMMs can easily capture complex system processes, such as those involving serial correlation, nonlinearity, non‐normality and non‐stationarity, in a tractable manner that goes well beyond the examples highlighted here. Instead of viewing these as a series of disparate domain‐specific applications of HMMs, we view them as a synthesis of the process by which ecologists can begin to critically think about their own sequential data, relate them to their particular system of interest and formulate an HMM for their specific domain using a simple conceptual template.

We foresee HMMs being more frequently used to integrate biotic and abiotic observations at large spatio‐temporal scales to investigate complex ecosystem‐level processes. The state process of the HMM could itself be at the ecosystem level (e.g. alternative stable states), or it could simply be used to account for unobservable state dynamics at lower levels of the hierarchy as a component of a larger (non‐Markovian) ecosystem‐level process model. Recent HMM methodological developments such as hierarchical formulations that allow data collection and/or state transitions to occur at multiple temporal resolutions (Fine *et al*., 1998; Leos‐Barajas *et al*., 2017a; Adam *et al*., 2019a), nonparametric approaches avoiding restrictive distributional assumptions (Yau *et al*., 2011; Langrock *et al*., 2018) and coupled HMMs for interacting state processes associated with different sequences (Sherlock *et al*., 2013; Touloupou *et al*., 2020) extend our capability to incorporate complex data structures and hierarchical relationships scaled from the individual to ecosystem level.

Despite this great potential, there remain several hurdles to the widespread implementation of HMMs describing long‐term, broad‐scale ecological dynamics (Turner *et al*., 1995; Lindenmayer *et al*., 2012; Haller, 2014). First, much like regression and analysis of variance, HMMs must become a familiar and accessible instrument within the ecologist's statistical ‘toolbox’. This has been the primary motivation for our review, and we hope our illustrative examples have provided a template by which researchers can begin to formulate HMMs according to their specific state and observation processes of interest. Second, although this challenge is by no means unique to HMMs, ecosystem‐level inferences continue to be limited by data availability, accessibility and compatibility (Jones *et al*., 2006; Dietze *et al*., 2018; Estes *et al*., 2018; Compagnoni *et al*., 2019; Halbritter *et al*., 2020), which can compromise our ability to empirically link observation and state processes operating at different spatio‐temporal scales. Third, as with any application of HMMs, such endeavours will require a faithful conceptualisation of ecosystem dynamics that is amenable to this discrete‐state modelling framework, as well as the identification and integration of observation processes that can provide information about the underlying system.

## Data Availability Statement

No new data were used.

## AUTHORSHIP

All authors conceived and wrote the manuscript.

### Peer Review

The peer review history for this article is available at https://publons.com/publon/10.1111/ele.13610.

## Supporting information

Supplementary Material

Supplementary Material
