# Supplementary material for: Uncovering ecological state dynamics with hidden Markov models
Source: Ecol Lett. 2020 Oct 19;23(12):1878–903. doi: 10.1111/ele.13610 (PMC7702077; doi:10.1111/ele.13610)
Supplement: Supplementary file 1 — Supplementary Material [file ELE-23-1878-s001.pdf]

Uncovering ecological state dynamics with hidden Markov models

—

Supplementary Material

Brett T. McClintock
NOAA National Marine Fisheries Service, U.S.A.
brett.mcclintock@noaa.gov

Roland Langrock
Department of Business Administration and Economics, Bielefeld University
roland.langrock@uni-bielefeld.de

Olivier Gimenez
CNRS Centre d'Ecologie Fonctionnelle et Evolutive, France
olivier.gimenez@cefe.cnrs.fr

Emmanuelle Cam
Laboratoire des Sciences de l'Environnement Marin, Institut Universitaire Européen de la Mer, Univ. Brest, CNRS, IRD, Ifremer, France
Emmanuelle.Cam@univ-brest.fr

David L. Borchers
School of Mathematics and Statistics, University of St Andrews
dlb@st-andrews.ac.uk

Richard Glennie
School of Mathematics and Statistics, University of St Andrews
rg374@st-andrews.ac.uk

Toby A. Patterson
CSIRO Oceans and Atmosphere, Australia
toby.patterson@csiro.au

Contents

Appendix A Dynamic species co-existence HMM **1**

Appendix B HMM software **3**

A Dynamic species co-existence HMM

Here we provide additional details of an HMM formulation for species co-existence dynamics based on presence-absence data (Marescot *et al.*, 2020). Let the states $S_t = \text{A}$ (respectively $S_t = \text{B}$ and $S_t = \text{AB}$) indicate "site occupied by species A" (respectively by species B and by both species) and $S_t = \text{U}$ indicate "unoccupied site". Define $X_{t,k} \in \{0, 1, 2, 3\}$, where 0 indicates neither species was detected, 1 indicates only species A was detected, 2 indicates only species B was detected, and 3 indicates both species were detected on the kth visit at time t. We could for example have:

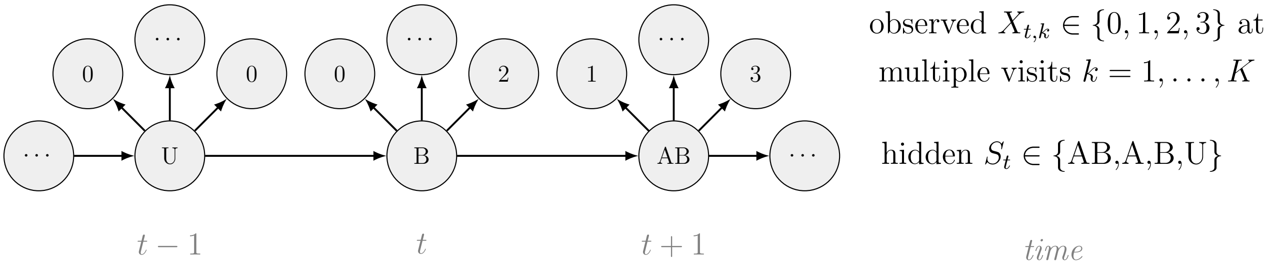

$$
\boldsymbol{\delta} = \begin{array}{c} \\ \left(\right. \end{array}
\begin{array}{cccc}
S_1 = \text{AB} & S_1 = \text{A} & S_1 = \text{B} & S_1 = \text{U} \\
\psi^{AB} & \psi^{A} & \psi^{B} & 1 - \psi^{AB} - \psi^{A} - \psi^{B}
\end{array}
\begin{array}{c} \\ \left. \right) \end{array}
$$

$$
\mathbf{\Gamma} = \begin{array}{c} \\ \left[\begin{array}{cccc} 1-\epsilon^{AB}-\epsilon^{A}+\epsilon^{B} & \epsilon^{B} & \epsilon^{A} & \epsilon^{AB} \\ \eta^{B} & 1-\omega^{A}-\eta^{B}-\nu^{A} & \omega^{A} & \nu^{A} \\ \eta^{A} & \omega^{B} & 1-\omega^{B}-\eta^{A}-\nu^{B} & \nu^{B} \\ \gamma^{AB} & \gamma^{A} & \gamma^{B} & 1-\gamma^{A}-\gamma^{B}-\gamma^{AB} \end{array}\right] \end{array}
\begin{array}{l} S_t = \text{AB} \\ S_t = \text{A} \\ S_t = \text{B} \\ S_t = \text{U} \end{array}
$$

(columns: $S_{t+1} = \text{AB}$, $S_{t+1} = \text{A}$, $S_{t+1} = \text{B}$, $S_{t+1} = \text{U}$)

with diagonal elements of $\mathbf{P}(\mathbf{x}_t)$

$$
\begin{aligned}
f(\mathbf{x}_t \mid S_t = \text{AB}) &= \prod_{k=1}^{K} r_{Ab}^{I(x_{t,k}=1)}(1-r_{Ab})^{1-I(x_{t,k}=1)} \\
&\quad + r_{aB}^{I(x_{t,k}=2)}(1-r_{aB})^{1-I(x_{t,k}=2)} \\
&\quad + r_{AB}^{I(x_{t,k}=3)}(1-r_{AB})^{1-I(x_{t,k}=3)} \\
f(\mathbf{x}_t \mid S_t = \text{A}) &= \prod_{k=1}^{K} p_{A}^{I(x_{t,k}=1)}(1-p_{A})^{1-I(x_{t,k}=1)} \\
f(\mathbf{x}_t \mid S_t = \text{B}) &= \prod_{k=1}^{K} p_{B}^{I(x_{t,k}=2)}(1-p_{B})^{1-I(x_{t,k}=2)} \\
f(\mathbf{x}_t \mid S_t = \text{U}) &= \prod_{k=1}^{K} I(x_{t,k}=0)
\end{aligned}
$$

where ψ_A (respectively ψ_B) is the probability of only species A (respectively B) being present, ψ_{AB} is the probability of both species being present, p_A (respectively p_B) is probability of detecting species A given only species A is present, r_{AB} is the probability of detecting both species given both species are present, r_{Ab} is the probability of detecting species A, not B, given both species are present, and r_{aB} is the probability of detecting species B, not A, given both species are present. The state transition probability matrix $\mathbf{\Gamma}$ is composed of the following parameters:

- ϵ_{AB} is the probability that both species A and B go locally extinct between t and $t+1$;
- ϵ_A (respectively ϵ_B) is the probability that species A goes locally extinct between t and

$t+1$, given both species are present at t;

- ν_A (respectively ν_B) is the probability that species A goes locally extinct between t and $t+1$, given species B was absent at t and $t+1$;
- γ_{AB} is the probability that both species A and B colonise a site between t and $t+1$;
- γ_A (respectively γ_B) is the probability that species A colonises a site between t and $t+1$, given both species are absent at t;
- η_A (respectively η_B) is the probability that species A colonises a site between t and $t+1$, given species B was present at t and $t+1$;
- ω_A (respectively ω_B) is the probability that species A is replaced by B between t and $t+1$.

B HMM software

The computational machinery of HMMs, such as the forward and Viterbi algorithms, can be coded from scratch by a proficient statistical programmer (e.g. Zucchini *et al.*, 2016; Louvrier *et al.*, 2018; Santostasi *et al.*, 2019), but recent advances in computing power and user-friendly software have made the implementation of HMMs much more feasible for practitioners. Many different HMM software packages and stand-alone programs are now available, some of which are focused on specific classes of state dynamics within the individual, population, or community level of the ecological hierarchy. However, the features and capabilities of the software are varied, and it can be challenging to determine which software may be most appropriate for a specific objective. Here we will describe some of the most popular HMM software currently available, including potential advantages and disadvantages for ecological applications. We limit our treatment to freely available R (R Core Team, 2019) packages and stand-alone programs that we believe are most accessible to ecologists and non-statisticians.

The Comprehensive R Archive Network (`https://cran.r-project.org`) currently hosts 26 packages that include "hidden Markov" in their description. While most HMM packages in R include data simulation, parameter estimation, and state decoding for an arbitrary number of system states, they differ in many key respects (see Table 2 in main text). Most of the packages are focused on categorical sequence analysis and are therefore limited in the state-dependent probability distributions that can be implemented (Himmelmann, 2010; Bartolucci *et al.*, 2017; Helske & Helske, 2019; Wilkinson, 2019). However, some of the more general packages provide greater flexibility for specifying state-dependent probability distributions, including commonly used discrete (e.g. binomial, Poisson) and continuous (e.g. gamma, normal) distributions (Visser & Speekenbrink, 2010; Jackson, 2011; Harte, 2017; McClintock & Michelot, 2018). One of the earliest and most flexible HMM packages, `depmixS4` (Visser & Speekenbrink, 2010), includes a broad range of probability distributions and can accommodate multivariate HMMs, multiple observation sequences (e.g. from multiple individuals or sites), parameter covariates, parameter constraints, and missing observations. With additional features originally motivated by animal movement HMMs (Michelot *et al.*, 2016), `momentuHMM` (McClintock & Michelot, 2018) is similar to `depmixS4` in terms of features and flexibility, but can also be used to implement mixed HMMs (DeRuiter *et al.*, 2017), hierarchical HMMs (Leos-Barajas *et al.*, 2017; Adam *et al.*, 2019), zero-inflated probability distributions (Martin *et al.*, 2005), and partially-observed state sequences. However, unlike `depmixS4` and other packages such as `mhsmm` (O'Connell & Højsgaard, 2011) and `HiddenMarkov` (Harte, 2017), `momentuHMM` does not currently support custom-coded state-dependent probability distributions. To our knowledge, only `hsmm` (Bulla & Bulla, 2013) and `mhsmm` (O'Connell & Højsgaard, 2011) can currently implement hidden semi-Markov models (Barbu & Limnios, 2009).

Many R packages are less general and specialise on specific HMM applications within individual- or population-level ecology. The `marked` package (Laake *et al.*, 2013) implements many of the popular capture-recapture HMMs described in Section 3.1. Packages that spe-

cialise in animal movement behaviour HMMs for telemetry data, such as those described in Section 3.1.2, include `bsam` (Jonsen *et al.*, 2005), `moveHMM` (Michelot *et al.*, 2016), and `momentuHMM` (McClintock & Michelot, 2018). The package `HMMoce` (Braun *et al.*, 2018) is specifically catered for HMMs that infer location from archival tag data (e.g. light levels, depth-temperature profiles) such as those described in Section 3.1.3. Using telemetry and count data, `kfdnm` (Schmidt *et al.*, 2015) can fit HMMs for population abundance and related demographic parameters such as those described in Section 3.2. The package `DDD` (Etienne & Haegeman, 2019) implements HMMs for macroevolutionary inference about diversification rates from phylogenetic trees such as those described in Section 3.2.2. The package `openpopscr` (Glennie *et al.*, 2019) can fit spatial capture-recapture HMMs that account for unobserved animal movements when estimating population-level density and survival, such as those described in Section 3.2.3. The popular package `unmarked` (Fiske & Chandler, 2011) includes many of the HMMs for inferring patterns and dynamics of species occurrence from repeated presence-absence data that were described in Section 3.2.3.

There are also several stand-alone, user-friendly software programs that focus on specific HMM applications in ecology. Programs `MARK` (White & Burnham, 1999) and `E-SURGE` (Choquet *et al.*, 2009) both provide a very general framework for implementing HMMs with individual-level capture-recapture (Pradel, 2005) or population-level presence-absence (Gimenez *et al.*, 2014) data, including observation process error arising from non-detection (Kellner & Swihart, 2014), state uncertainty (Kendall, 2009; Kendall *et al.*, 2012), and species misidentfication (Miller *et al.*, 2011). Program `PRESENCE` (Hines, 2006) has many of the features of `MARK` and `E-SURGE` but focuses solely on presence-absence data, including models for species co-occurrence dynamics (MacKenzie *et al.*, 2018). The `RMark` (Laake, 2013) and `RPresence` (MacKenzie & Hines, 2018) packages have been developed as R interfaces for Programs `MARK` and `PRESENCE`, respectively.

Although not intended specifically for HMMs, it is worth noting that there are a number of

software programs with which these types of models can be relatively easily implemented by users with minimal statistical programming experience. For Bayesian inference using MCMC sampling (Gelman *et al.*, 2004), these include `WinBUGS`/`OpenBUGS` (Lunn *et al.*, 2009; Kéry & Schaub, 2011; Lunn *et al.*, 2012), `JAGS` (Plummer, 2003), and `Stan` (Gelman *et al.*, 2015). There are R package interfaces for all of these programs, including `R2OpenBUGS` (Sturtz *et al.*, 2005), `rjags` (Plummer, 2019), and `rstan` (Stan Development Team, 2019), respectively. The R package `nimble` (de Valpine *et al.*, 2017) and its `nimbleEcology` extension for common HMMs in ecology (Goldstein *et al.*, 2019) use a statistical programming language similar to `BUGS` and can be used for Bayesian or maximum likelihood inference. The R package `pomp` (King *et al.*, 2016) specialises in general state-space models (including HMMs) and can also be used for Bayesian or maximum likelihood inference. Focusing on Bayesian state-space models, the package `rbi` is a complete R interface for the `LibBi` library (Murray, 2015). The R package `TMB` (Kristensen *et al.*, 2016) generally has a steeper learning curve but can be advantageous for maximum likelihood inference (e.g. Benhaiem *et al.*, 2018; Marescot *et al.*, 2018), particularly for mixed HMMs that include continuous-valued random effects (Altman, 2007). From a computational point of view, neither maximum likelihood estimation nor MCMC sampling is vastly superior (cf. Patterson *et al.*, 2017, for a more comprehensive discussion). However, MCMC samplers that include both the parameter vector ($\boldsymbol{\theta}$) and the latent states ($S_1, \ldots, S_T$), as commonly implemented in `WinBUGS`/`OpenBUGS` and `JAGS`, are inherently slow; sampling from the parameter vector only while applying the forward algorithm to evaluate the likelihood will often be preferable (Turek *et al.*, 2016; Yackulic *et al.*, 2020).

References

Adam, T., Griffiths, C. A., Leos-Barajas, V., Meese, E. N., Lowe, C. G., Blackwell, P. G., Righton, D. & Langrock, R. (2019). Joint modelling of multi-scale animal movement data

using hierarchical hidden Markov models. *Methods in Ecology and Evolution*, 10, 1536–1550.

Altman, R. M. (2007). Mixed hidden Markov models: an extension of the hidden Markov model to the longitudinal data setting. *Journal of the American Statistical Association*, 102, 201–210.

Barbu, V. S. & Limnios, N. (2009). *Semi-Markov Chains and Hidden Semi-Markov Models Toward Applications: Their Use in Reliability and DNA Analysis*, vol. 191 of *Lecture Notes in Statistics*. Springer.

Bartolucci, F., Pandolfi, S. & Pennoni, F. (2017). LMest: An R package for latent Markov models for longitudinal categorical data. *Journal of Statistical Software*, 81, 1–38.

Benhaiem, S., Marescot, L., Hofer, H., East, M. L., Lebreton, J.-D., Kramer-Schadt, S. & Gimenez, O. (2018). Robustness of eco-epidemiological capture-recapture parameter estimates to variation in infection state uncertainty. *Frontiers in Veterinary Science*, 5, 197.

Braun, C. D., Galuardi, B. & Thorrold, S. R. (2018). Hmmoce: An r package for improved geolocation of archival-tagged fishes using a hidden Markov method. *Methods in Ecology and Evolution*, 9, 1212–1220.

Bulla, J. & Bulla, I. (2013). *hsmm: Hidden Semi Markov Models*. URL https://CRAN.R-project.org/package=hsmm. R package version 0.4.

Choquet, R., Rouan, L. & Pradel, R. (2009). Program E-SURGE: a software application for fitting multievent models. In: *Modeling demographic processes in marked populations* (eds. Thomson, D. L., Cooch, E. G. & Conroy, M. J.). Springer, pp. 845–865.

de Valpine, P., Turek, D., Paciorek, C. J., Anderson-Bergman, C., Lang, D. T. & Bodik, R. (2017). Programming with models: writing statistical algorithms for general model structures with NIMBLE. *Journal of Computational and Graphical Statistics*, 26, 403–413.

DeRuiter, S. L., Langrock, R., Skirbutas, T., Goldbogen, J. A., Calambokidis, J., Friedlaender, A. S. & Southall, B. L. (2017). A multivariate mixed hidden Markov model to analyze blue whale diving behaviour during controlled sound exposures. *The Annals of Applied Statistics*, 11, 362–392.

Etienne, R. S. & Haegeman, B. (2019). *DDD: Diversity-Dependent Diversification*. URL https://CRAN.R-project.org/package=DDD. R package version 4.1.

Fiske, I. & Chandler, R. (2011). unmarked: an R package for fitting hierarchical models of wildlife occurrence and abundance. *Journal of Statistical Software*, 43, 1–23.

Gelman, A., Carlin, J. B., Stern, H. S. & Rubin, D. B. (2004). *Bayesian Data Analysis, 2nd Edition*. Chapman and Hall, Boca Raton.

Gelman, A., Lee, D. & Guo, J. (2015). Stan: A probabilistic programming language for bayesian inference and optimization. *Journal of Educational and Behavioral Statistics*, 40, 530–543.

Gimenez, O., Blanc, L., Besnard, A., Pradel, R., Doherty Jr, P. F., Marboutin, E. & Choquet, R. (2014). Fitting occupancy models with E-SURGE: hidden Markov modelling of presence–absence data. *Methods in Ecology and Evolution*, 5, 592–597.

Glennie, R., Borchers, D. L., Murchie, M., Harmsen, B. J. & Foster, R. J. (2019). Open population maximum likelihood spatial capture-recapture. *Biometrics*, 75, 1345–1355.

Goldstein, B. R., Turek, D., Ponisio, L. & de Valpine, P. (2019). *nimbleEcology: Distributions for Ecological Models in 'nimble'*. URL https://CRAN.R-project.org/package=nimbleEcology. R package version 0.1.0.

Harte, D. (2017). *HiddenMarkov: Hidden Markov Models*. Statistics Research Associates,

Wellington. URL http://www.statsresearch.co.nz/dsh/sslib/. R package version 1.8-11.

Helske, S. & Helske, J. (2019). Mixture hidden Markov models for sequence data: The seqHMM package in R. *Journal of Statistical Software*, 88, 1–32.

Himmelmann, L. (2010). *HMM: Hidden Markov Models*. URL https://CRAN.R-project.org/package=HMM. R package version 1.0.

Hines, J. E. (2006). *PRESENCE2 - Software to estimate patch occupancy and related parameters*. URL http://www.mbr-pwrc.usgs.gov/software/presence.html. USGS–PWRC.

Jackson, C. H. (2011). Multi-state models for panel data: the msm package for R. *Journal of statistical software*, 38, 1–29.

Jonsen, I. D., Flemming, J. M. & Myers, R. A. (2005). Robust state–space modeling of animal movement data. *Ecology*, 86, 2874–2880.

Kellner, K. F. & Swihart, R. K. (2014). Accounting for imperfect detection in ecology: a quantitative review. *PLoS ONE*, 9, e111436.

Kendall, W. L. (2009). One size does not fit all: adapting mark-recapture and occupancy models for state uncertainty. In: *Modeling demographic processes in marked populations*. Springer, pp. 765–780.

Kendall, W. L., White, G. C., Hines, J. E., Langtimm, C. A. & Yoshizaki, J. (2012). Estimating parameters of hidden Markov models based on marked individuals: use of robust design data. *Ecology*, 93, 913–920.

Kéry, M. & Schaub, M. (2011). *Bayesian Population Analysis Using WinBUGS: a Hierarchical Perspective*. Academic Press.

King, A. A., Nguyen, D. & Ionides, E. L. (2016). Statistical inference for partially observed Markov processes via the R package pomp. *Journal of Statistical Software*, 69, 1–43.

Kristensen, K., Nielsen, A., Berg, C. W., Skaug, H. J. & Bell, B. (2016). Tmb: Automatic differentiation and laplace approximation. *Journal of Statistical Software*, 70, 1–21.

Laake, J. L. (2013). *RMark: An R Interface for Analysis of Capture-Recapture Data with MARK*. AFSC Processed Rep 2013-01, 25p. Alaska Fish. Sci. Cent., NOAA, Natl. Mar. Fish. Serv., 7600 Sand Point Way NE, Seattle WA 98115.

Laake, J. L., Johnson, D. S. & Conn, P. B. (2013). marked: an R package for maximum likelihood and Markov chain Monte Carlo analysis of capture–recapture data. *Methods in Ecology and Evolution*, 4, 885–890.

Leos-Barajas, V., Gangloff, E. J., Adam, T., Langrock, R., Van Beest, F. M., Nabe-Nielsen, J. & Morales, J. M. (2017). Multi-scale modeling of animal movement and general behavior data using hidden Markov models with hierarchical structures. *Journal of Agricultural, Biological and Environmental Statistics*, 22, 232–248.

Louvrier, J., Chambert, T., Marboutin, E. & Gimenez, O. (2018). Accounting for misidentification and heterogeneity in occupancy studies using hidden Markov models. *Ecological Modelling*, 387, 61–69.

Lunn, D., Jackson, C., Best, N., Spiegelhalter, D. & Thomas, A. (2012). *The BUGS book: A practical introduction to Bayesian analysis*. Chapman and Hall/CRC.

Lunn, D., Spiegelhalter, D., Thomas, A. & Best, N. (2009). The bugs project: Evolution, critique and future directions. *Statistics in medicine*, 28, 3049–3067.

MacKenzie, D. & Hines, J. (2018). *RPresence: R Interface for Program PRESENCE*. URL https://www.mbr-pwrc.usgs.gov/software/presence.html. R package version 2.12.34.

MacKenzie, D. I., Nichols, J. D., Royle, J. A., Pollock, K. H., Bailey, L. & Hines, J. E. (2018). *Occupancy Estimation and Modeling: Inferring Patterns and Dynamics of Species Occurrence*. 2nd edn. Elsevier.

Marescot, L., Benhaiem, S., Gimenez, O., Hofer, H., Lebreton, J.-D., Olarte-Castillo, X. A., Kramer-Schadt, S. & East, M. L. (2018). Social status mediates the fitness costs of infection with canine distemper virus in Serengeti spotted hyenas. *Functional Ecology*, 32, 1237–1250.

Marescot, L., Lyet, A., Singh, R., Carter, N. & Gimenez, O. (2020). Inferring wildlife poaching in southeast Asia with multispecies dynamic occupancy models. *Ecography*, 43, 239–250.

Martin, T. G., Wintle, B. A., Rhodes, J. R., Kuhnert, P. M., Field, S. A., Low-Choy, S. J., Tyre, A. J. & Possingham, H. P. (2005). Zero tolerance ecology: improving ecological inference by modelling the source of zero observations. *Ecology Letters*, 8, 1235–1246.

McClintock, B. T. & Michelot, T. (2018). momentuHMM: R package for generalized hidden Markov models of animal movement. *Methods in Ecology and Evolution*, 9, 1518–1530.

Michelot, T., Langrock, R. & Patterson, T. A. (2016). moveHMM: An R package for the statistical modelling of animal movement data using hidden Markov models. *Methods in Ecology and Evolution*, 7, 1308–1315.

Miller, D. A., Nichols, J. D., McClintock, B. T., Grant, E. H. C., Bailey, L. L. & Weir, L. A. (2011). Improving occupancy estimation when two types of observational error occur: non-detection and species misidentification. *Ecology*, 92, 1422–1428.

Murray, L. (2015). Bayesian state-space modelling on high-performance hardware using libbi. *Journal of Statistical Software, Articles*, 67, 1–36.

O'Connell, J. & Højsgaard, S. (2011). Hidden semi Markov models for multiple observation sequences: The mhsmm package for R. *Journal of Statistical Software*, 39, 1–22.

Patterson, T. A., Parton, A., Langrock, R., Blackwell, P. G., Thomas, L. & King, R. (2017). Statistical modelling of individual animal movement: an overview of key methods and a discussion of practical challenges. *AStA Advances in Statistical Analysis*, 101, 399–438.

Plummer, M. (2003). JAGS: A program for analysis of Bayesian graphical models using Gibbs sampling. In: *Proceedings of the 3rd international workshop on distributed statistical computing*, vol. 124. Vienna, Austria.

Plummer, M. (2019). *rjags: Bayesian Graphical Models using MCMC*. URL https://CRAN.R-project.org/package=rjags. R package version 4-9.

Pradel, R. (2005). Multievent: An extension of multistate capture-recapture models to uncertain states. *Biometrics*, 61, 442–447.

R Core Team (2019). *R: A Language and Environment for Statistical Computing*. R Foundation for Statistical Computing, Vienna, Austria. URL https://www.R-project.org/.

Santostasi, N. L., Ciucci, P., Caniglia, R., Fabbri, E., Molinari, L., Reggioni, W. & Gimenez, O. (2019). Use of hidden Markov capture–recapture models to estimate abundance in the presence of uncertainty: Application to the estimation of prevalence of hybrids in animal populations. *Ecology and evolution*, 9, 744–755.

Schmidt, J. H., Johnson, D. S., Lindberg, M. S. & Adams, L. G. (2015). Estimating demographic parameters using a combination of known-fate and open N-mixture models. *Ecology*, 96, 2583–2589.

Stan Development Team (2019). RStan: the R interface to Stan. URL http://mc-stan.org/. R package version 2.19.2.

Sturtz, S., Ligges, U. & Gelman, A. (2005). R2WinBUGS: A package for running WinBUGS from R. *Journal of Statistical Software*, 12, 1–16.

Turek, D., de Valpine, P. & Paciorek, C. J. (2016). Efficient Markov chain Monte Carlo sampling for hierarchical hidden Markov models. *Environmental and Ecological Statistics*, 23, 549–564.

Visser, I. & Speekenbrink, M. (2010). depmixS4: an R package for hidden Markov models. *Journal of Statistical Software*, 36, 1–21.

White, G. C. & Burnham, K. P. (1999). Program MARK: Survival estimation from populations of marked animals. *Bird Study*, 46, S120–S138.

Wilkinson, S. (2019). aphid: an R package for analysis with profile hidden Markov models. *Bioinformatics*, 35, 3829–3830.

Yackulic, C. B., Dodrill, M., Dzul, M., Sanderlin, J. S. & Reid, J. A. (2020). A need for speed in Bayesian population models: a practical guide to marginalizing and recovering discrete latent states. *Ecological Applications*, 30, e02112.

Zucchini, W., MacDonald, I. L. & Langrock, R. (2016). *Hidden Markov Models for Time Series: An Introduction Using R*. 2nd edn. CRC Press.
