# Supplementary material for: Uncovering ecological state dynamics with hidden Markov models
Source: Ecol Lett. 2020 Oct 19;23(12):1878–903. doi: 10.1111/ele.13610 (PMC7702077; doi:10.1111/ele.13610)
Supplement: Supplementary file 2 — Supplementary Material [file ELE-23-1878-s002.pdf]

Uncovering ecological state dynamics with hidden Markov models

—

Supplementary Tutorial

Brett T. McClintock
NOAA National Marine Fisheries Service, U.S.A.
brett.mcclintock@noaa.gov

Roland Langrock
Department of Business Administration and Economics, Bielefeld University
roland.langrock@uni-bielefeld.de

Olivier Gimenez
CNRS Centre d'Ecologie Fonctionnelle et Evolutive, France
olivier.gimenez@cefe.cnrs.fr

Emmanuelle Cam
Laboratoire des Sciences de l'Environnement Marin, Institut Universitaire Européen de la Mer, Univ. Brest, CNRS, IRD, Ifremer, France
Emmanuelle.Cam@univ-brest.fr

David L. Borchers
School of Mathematics and Statistics, University of St Andrews
dlb@st-andrews.ac.uk

Richard Glennie
School of Mathematics and Statistics, University of St Andrews
rg374@st-andrews.ac.uk

Toby A. Patterson
CSIRO Oceans and Atmosphere, Australia
toby.patterson@csiro.au

Contents

S1 Introduction 1

S2 Building the HMM 3

S2.1 State process 3

S2.2 Observation process 6

S3 Parameter estimation 7

S3.1 Overview 7

S3.2 Likelihood evaluation using the forward algorithm 7

S3.3 Model fitting 10

S4 Model selection & model checking 13

S4.1 Model selection 13

S4.2 Model checking 14

S5 State decoding 17

S5.1 Overview 17

S5.2 Computation 18

S6 Interpretation 20

S1 Introduction

In the main text, we review how hidden Markov models (HMMs) are used to uncover ecological dynamics that operate at the individual, population, community, and ecosystem levels. The breadth of application of HMMs in ecology shows that their general structure can reflect ecological processes or how we understand and summarise ecological processes. Nevertheless,

formulating an HMM that faithfully represents the ecological dynamics under study, fitting this HMM to real data, and checking that the HMM is a good model to use are non-trivial steps in any analysis. In this supplementary tutorial, we introduce the main steps in an HMM analysis, highlighting the decisions and assumptions to be considered along the way.

As demonstrated in the main text, each application of an HMM is particular to the problem under study. Here we illustrate how to tailor an HMM to a given study, thereby showcasing the inferential tools available for HMMs. We consider a single application, introduced in the main text: a time series of the vectorial sum of the dynamic body acceleration (VDBA) of a striated caracara (*Phalcoboenus australis*) measured every second over one hour (Fahlbusch and Harrington, 2019). VDBA is a measure of the overall activity of an animal: it is the length of the three-dimensional vector defined by the dynamic accelerations in all three coordinate directions (Qasem et al., 2012). Figure 1 shows the VDBA time series considered here.

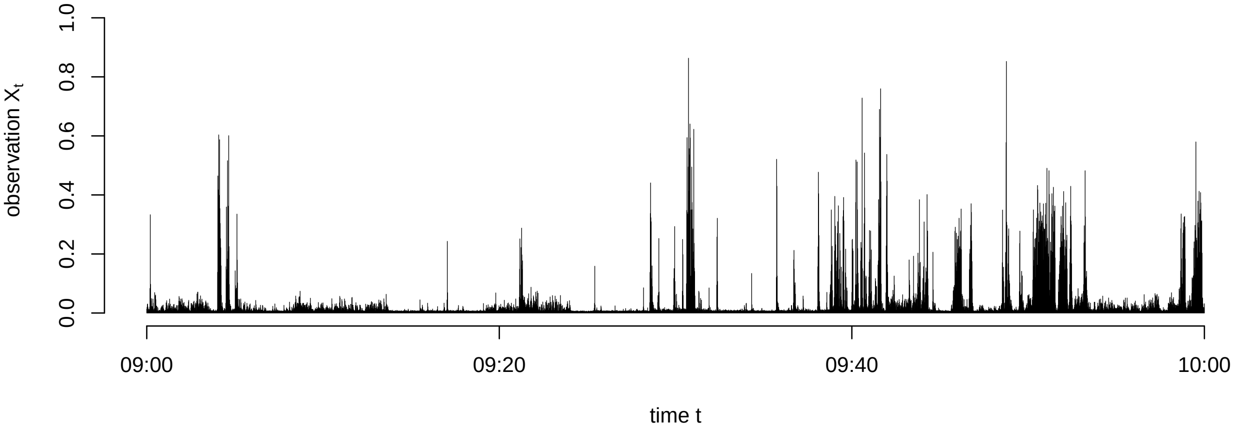

Figure 1: Vectorial dynamic body acceleration X_t, $t = 1, \ldots, 3600$, for a striated caracara (*Phalcoboenus australis*) measured over one hour at 1 Hz (Fahlbusch and Harrington, 2019).

We consider the full analysis cycle, comprising the formulation of an HMM, the estimation of its parameters, the selection between different candidate models, checking how well the model fits the data, inferring the latent states, and interpreting the final results. The supplemental R (R Core Team, 2019) script `caracaraExample.R` can be used to completely

reproduce and further explore this illustrative example using the package `momentuHMM` (McClintock and Michelot, 2018).

S2 Building the HMM

The observations are realisations of the sequential process $X_1, \ldots, X_T$, often a time series, which we call the observation process (or state-dependent process). HMMs comprise two parts: a model for the observation process and a model for the state process underlying the observation process. Each part has associated *assumptions* which induce both the simplicity that HMMs are favoured for and the limitations that may inhibit their ability to faithfully characterise either process.

S2.1 State process

The state process $S_1, \ldots, S_T$ is modelled as a Markov chain over a set of N states, i.e. $S_t \in \{1, \ldots, N\}$ for $t = 1, \ldots, T$. The key assumption involved here is that the state process is Markovian: the probability of which state occurs next in time is known conditional on the current state, irrespective of the states that occurred in the past. As a consequence, the way the process evolves over time is completely determined by the $N \times N$ state transition probability matrix, which comprises the probabilities of switching from any state i at time t to any state j at time $t + 1$, for $i, j = 1, \ldots, N$. The Markov property also implies that the time spent in each state has a geometric distribution. Clearly, this is a strong assumption on the memory of the ecological process and so it is important to remember this specification in light of interpreting results.

Specifying the state process involves selecting the number of states as well as any potential pre-determined structure in the transition probability matrix. To make either of these decisions, one must first consider what latent ecological process drives the observations and

whether such a process, or a simplified version of it, can be described by a Markov chain taking finitely many states. The idea behind specifying the model for the state process is to create a structure that is likely to capture the pattern in the underlying ecological dynamics that the observations evince, with the caveat that beyond any pre-defined structure the state characteristics are driven solely by data and may, in the end, not necessarily conform with our original intentions for them.

For the VDBA example, we must rely on our understanding of the movement of the study species. It is common to consider the activity level of an animal to vary across different behavioural modes (see Section 3.1.3 in the main text). The model states are thus intended to correspond with these behaviours. Whereas in some applications of HMMs the choice of the number of states can be obvious, such as in the Cormack-Jolly-Seber model with the two states "alive" and "dead" (see Section 3.1.1 in the main text), there are also such where it is not clear *a priori* how many states there are. This is indeed the case in the caracara example considered here. In cases such as these the choice of N should be guided by expert knowledge on the subject matter. In the given VDBA example, the research biologists who collected the data expected $N = 4$ states, corresponding to resting behaviour, minimal activity such as preening, moderate activity such as walking or digging, and flight, to most adequately reflect the variation in activity. In general, selecting the maximum number of states to consider is a trade-off between computational feasibility, ecological knowledge of the system, and fit to the data. Pohle et al. (2017) provide a step-by-step guide to selecting the number of states for an HMM and discuss why relying solely on statistical model selection methods, such as the Akaike information criterion (AIC) or the Bayesian information criterion (BIC), may not be desirable.

Along with the number of states, we specify that it is possible for the animal to switch from any one behaviour to any other and so the transition probability matrix has unknown

parameters for all entries:

$$\boldsymbol{\Gamma} = \begin{array}{c} \\ \left[\begin{array}{cccc} \gamma_{1,1} & \gamma_{1,2} & \gamma_{1,3} & \gamma_{1,4} \\ \gamma_{2,1} & \gamma_{2,2} & \gamma_{2,3} & \gamma_{2,4} \\ \gamma_{3,1} & \gamma_{3,2} & \gamma_{3,3} & \gamma_{3,4} \\ \gamma_{4,1} & \gamma_{4,2} & \gamma_{4,3} & \gamma_{4,4} \end{array} \right] \end{array} \begin{array}{l} S_t = 1 \\ S_t = 2 \\ S_t = 3 \\ S_t = 4 \end{array} .$$

(columns: $S_{t+1} = 1$, $S_{t+1} = 2$, $S_{t+1} = 3$, $S_{t+1} = 4$)

Further to this, we specify the distribution of the states at time $t = 1$, hence the probabilities $\delta_i = \Pr(S_1 = i)$ of the animal inhabiting any state i, $i = 1, \ldots, N$, at the start of the sequence, to be the stationary distribution of the Markov chain (see Zucchini et al., 2016, p. 17).

This is the most general transition probability matrix to consider. In other contexts, it can be appropriate to limit transitions between states. For example, in Cormack-Jolly-Seber models the states are intended to represent "alive" and "dead", so it is necessary to forbid transitions from the "dead" to "alive" state by enforcing a zero value in the corresponding entry of $\boldsymbol{\Gamma}$. Additional structure, such as temporal heterogeneity in the transition probability matrix ($\boldsymbol{\Gamma}_t$), can also be incorporated through the use of explanatory covariates (e.g. Morales et al., 2004; Li and Bolker, 2017). This is usually accomplished using a multinomal-logit link function:

$$\gamma_{t,i,j} = \frac{\exp\left(\beta_{0,i,j} + \sum_{k=1}^{K} z_{k,t}\beta_{k,i,j}\right)}{\sum_{l=1}^{N} \exp\left(\beta_{0,i,l} + \sum_{k=1}^{K} z_{k,t}\beta_{k,i,l}\right)},$$

where $\gamma_{t,i,j}$ is the time-dependent transition probability from state i at time t to state j at time $t+1$, $\beta_{0,i,j}$ is an intercept term, $z_{k,t}$ is the value of the kth covariate at time t, and $\beta_{k,i,j}$ is a slope term for the kth covariate. Because $\sum_{j=1}^{N} \gamma_{t,i,j} = 1$, it is customary to set $\alpha_{i,j} = \beta_{k,i,j} = 0$ for $i = j$ and $k = 1, \ldots, K$ to avoid overparameterisation when estimating these parameters (see Section S3). This extension is not implemented in the given example, but see White and Burnham (1999), MacKenzie et al. (2002), and Patterson et al. (2009) for

common examples of link functions in HMMs.

S2.2 Observation process

Conditional on the state process, each observation arises as a sample from a distribution — selected from N possible distributions according to the current underlying state — that is unrelated to previous states or observations. This assumption of the observations being conditionally independent of each other, given the states, is again a strong assumption and essentially forces all the serial dependence in the observations to be described by the state process alone, leaving the state-dependent distributions to capture the commonality between observations within each state, independent of time. In particular, for any period of time that the state process remains within a single state, the observations within that period are assumed to be independent of each other, which will not always be realistic.

HMMs are extremely flexible with respect to observation type: observations can be discrete or continuous, multivariate, and have distributions with parameters that depend on covariates via suitable link functions (e.g. see Table 2 in McClintock and Michelot, 2018). As with the state process, the observation process can include additional pre-defined structure that reflects the system of interest. For example, in Cormack-Jolly-Seber models any individuals in the "dead" state cannot be detected, and the state-dependent distribution for the "dead" state is therefore pre-defined as a Bernoulli distribution with a success probability of zero (see Section 3.1.1 in the main text).

In the striated caracara example, VDBA is a positive and continuous quantity. It therefore seems adequate to use for example Weibull or gamma state-dependent distributions in this case. Below we show the results obtained assuming VDBA at time t (X_t) to be gamma-distributed within each state:

$$X_t \mid S_t = i \sim \text{Gamma}\left(\kappa_i, \rho_i\right),$$

where κ_i and ρ_i are the shape and scale parameters, respectively, of the state-dependent gamma distribution for $i \in \{1, 2, 3, 4\}$, with corresponding mean $\mu_i = \kappa_i \rho_i$ and variance $\sigma_i^2 = \kappa_i \rho_i^2$. We have imposed no additional structure for the VDBA observations, and all of the state-dependent parameters are therefore to be freely estimated and entirely data-driven.

S3 Parameter estimation

S3.1 Overview

Once a model formulation has been identified, the next step is to estimate the model's parameters, summarised in the parameter vector $\boldsymbol{\theta}$, based on the observation sequence $(x_1, \ldots, x_T)$. There are three main strategies for estimating the parameters of an HMM:

- maximum likelihood by direct numerical maximisation of the likelihood;
- maximum likelihood by the expectation-maximisation (EM) algorithm;
- Bayesian inference using Markov chain Monte Carlo (MCMC).

Accessible reviews on the theoretical and practical differences between maximum likelihood and Bayesian analysis methods include Ellison (2004), Newman et al. (2014, Chapter 4), and Patterson et al. (2017). Although not intended specifically for ecologists, Rabiner (1989) provides a very accessible introduction to fitting HMMs using the EM algorithm. Within each of these approaches, the existence of efficient recursive schemes for evaluating the likelihood of an HMM is a key asset. We therefore proceed by providing detailed information on how the likelihood is evaluated.

S3.2 Likelihood evaluation using the forward algorithm

Using the model assumptions — i.e. the Markov property for the state process $S_1, S_2, \ldots, S_T$, and conditional independence of the observations $X_1, X_2, \ldots, X_T$, given the states — the

likelihood of an N–state HMM can be obtained as

$$\begin{aligned}\mathcal{L}(\boldsymbol{\theta} \mid x_1, \dots, x_T) &= f_{\boldsymbol{\theta}}(x_1, \dots, x_T) \\ &= \sum_{s_1=1}^{N} \dots \sum_{s_T=1}^{N} f_{\boldsymbol{\theta}}(x_1, \dots, x_T | s_1, \dots, s_T) f_{\boldsymbol{\theta}}(s_1, \dots, s_T) \\ &= \sum_{s_1=1}^{N} \dots \sum_{s_T=1}^{N} \delta_{s_1} \prod_{t=1}^{T} f_{\boldsymbol{\theta}}(x_t | s_t) \prod_{t=2}^{T} \gamma_{s_{t-1}, s_t}.\end{aligned}$$

The first step uses the law of total probability, while the second is an immediate consequence of the model's dependence structure. The state-dependent densities (or probabilities, for discrete data) $f_{\boldsymbol{\theta}}(x_t|s_t)$ depend on the distributional assumption made for the state-dependent (observation) process. In the VDBA example continued from above, with our assumptions made these would be densities of the gamma distribution, with one set of shape and scale parameters for each of the N possible states.

In this form, the likelihood involves N^T summands, rendering its evaluation infeasible even for a moderate number of observations, T. This has led some users to believe that standard (numerical) likelihood maximisation is not feasible for HMMs. This is generally not true, as there is in fact a much more efficient way to calculcate the likelihood, namely the *forward algorithm*, which exploits the model's dependence structure to avoid the above brute force summation over all possible state sequences. To explain the inner workings of the forward algorithm, we define the forward variables at time t as

$$\alpha_t(j) = f_{\boldsymbol{\theta}}(x_1, ..., x_t, s_t = j), \quad j = 1, \dots, N,$$

which can be summarised in a vector as $\boldsymbol{\alpha}_t = \big(\alpha_t(1), \dots, \alpha_t(N)\big)$. A close look at $\alpha_t(j)$ reveals that this quantity comprises information on both the likelihood of all observations up to time

t, since

$$f(x_1, ..., x_t) = \sum_{j=1}^{N} f(x_1, ..., x_t, s_t = j) = \sum_{j=1}^{N} \alpha_t(j),$$

as well as on the conditional probability of state j being active at time t, given all observations up to time t, since

$$\Pr(S_t = j \mid x_1, \dots, x_t) = \frac{f(x_1, ..., x_t, s_t = j)}{f(x_1, ..., x_t)} = \frac{\alpha_t(j)}{\sum_{k=1}^{N} \alpha_t(k)}.$$

The forward algorithm now traverses along the time series, updating $\boldsymbol{\alpha}_t$ step-by-step (and hence the likelihood, while retaining information on the probabilities of being in the different states). More specifically, from the dependence assumptions it follows that

$$\alpha_t(j) = \sum_{i=1}^{N} \alpha_{t-1}(i) \gamma_{ij} f_{\boldsymbol{\theta}}(x_t \mid s_t = j).$$

In matrix notation, this becomes

$$\boldsymbol{\alpha}_t = \boldsymbol{\alpha}_{t-1} \boldsymbol{\Gamma} \mathbf{P}(x_t),$$

where $\mathbf{P}(x_t) = \text{diag}\big(f_{\boldsymbol{\theta}}(x_t \mid s_t = 1), \dots, f_{\boldsymbol{\theta}}(x_t \mid s_t = N)\big)$. Together with the initial calculation $\boldsymbol{\alpha}_1 = \boldsymbol{\delta} \mathbf{P}(x_1)$, this is the forward algorithm. Note that the exact specification of three model-defining components, $\boldsymbol{\delta}$ (initial state distribution), $\boldsymbol{\Gamma}$ (state transition probability matrix), and $\mathbf{P}(x_t)$ (state-dependent observation distributions), depends on the model formulation considered — as shown in the many detailed examples provided in the main text as well as the specific example of the VDBA series described above.

The forward algorithm can be applied in order to first calculate $\boldsymbol{\alpha}_1$, then $\boldsymbol{\alpha}_2$, etc., until one arrives at $\boldsymbol{\alpha}_T$, the sum of all elements of which obviously yields the likelihood. Thus,

$$\mathcal{L}(\boldsymbol{\theta} \mid x_1, \dots, x_T) = \boldsymbol{\delta} \mathbf{P}(x_1) \boldsymbol{\Gamma} \mathbf{P}(x_2) \cdots \boldsymbol{\Gamma} \mathbf{P}(x_{T-1}) \boldsymbol{\Gamma} \mathbf{P}(x_T) \mathbf{1}\,, \tag{1}$$

where **1** is a column vector of ones. The computational complexity of evaluating Eq. (1) is $\mathcal{O}(TN^2)$, meaning it is *linear* in the number of observations, T, such that likelihood evaluation is typically feasible even for sequences comprising millions of observations. The existence of such recursive techniques for fast evaluation of the likelihood is a key reason for the popularity of HMMs. It is worth pointing out that this step-by-step recursive calculation is possible due to the dependence assumptions made at the process.

S3.3 Model fitting

Of the three common approaches to fitting an HMM — numerical likelihood maximisation, EM algorithm, and MCMC — we used numerical likelihood maximisation for the VDBA data. For example with $N = 4$ states, we obtain estimates for the initial distribution, transition probability matrix, and state-dependent distributions (Figure 2):

$$\begin{array}{cc} & \begin{array}{cccc} S_1 = 1 & S_1 = 2 & S_1 = 3 & S_1 = 4 \end{array} \\ \hat{\boldsymbol{\delta}} = & \begin{bmatrix} 0.30 & 0.28 & 0.28 & 0.14 \end{bmatrix} \end{array}$$

$$\begin{array}{ccc} & \begin{array}{cccc} S_{t+1} = 1 & S_{t+1} = 2 & S_{t+1} = 3 & S_{t+1} = 4 \end{array} & \\ \widehat{\boldsymbol{\Gamma}} = & \begin{bmatrix} 0.90 & 0.09 & 0.00 & 0.00 \\ 0.1 & 0.73 & 0.16 & 0.01 \\ 0.00 & 0.17 & 0.79 & 0.04 \\ 0.00 & 0.00 & 0.10 & 0.90 \end{bmatrix} & \begin{array}{c} S_t = 1 \\ S_t = 2 \\ S_t = 3 \\ S_t = 4 \end{array} \end{array}$$

$$\begin{array}{cc} & \begin{array}{cccc} S_t = 1 & S_t = 2 & S_t = 3 & S_t = 4 \end{array} \\ \hat{\boldsymbol{\mu}} = & \begin{bmatrix} 0.007 & 0.013 & 0.032 & 0.227 \end{bmatrix} \end{array}$$

$$\begin{array}{ccccc} & S_t = 1 & S_t = 2 & S_t = 3 & S_t = 4 \\ \hat{\boldsymbol{\sigma}} = & \Big[\, 0.001 & 0.004 & 0.014 & 0.132 \,\Big] \end{array}$$

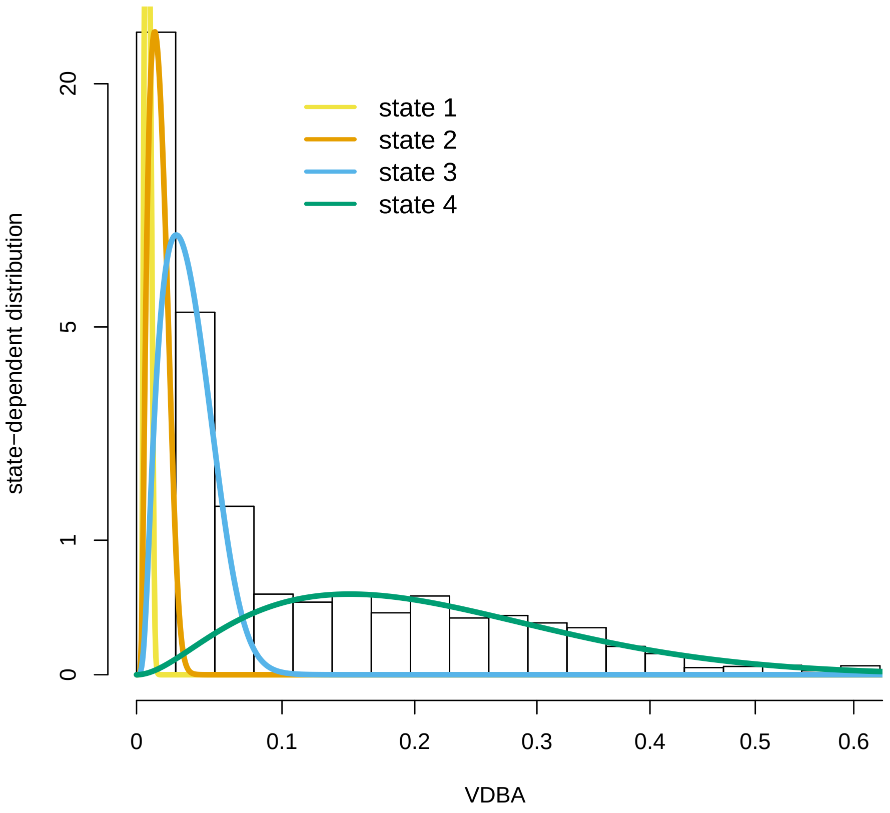

Figure 2: Estimated gamma state-dependent distributions within a four-state HMM for a striated caracara.

The relatively low computational cost involved in evaluating the likelihood renders numerical maximisation feasible in most cases. For example, fitting the HMM above with $N = 4$ states to the VDBA sequence comprising 3600 observations took 45 seconds in R on an octa-core i7 CPU, at 3.4 GHz and with 8 GB RAM — analysing sequences comprising millions of observations is also generally feasible (depending on model complexity). The computation

time can often further substantially be reduced by implementing the forward algorithm in C++, as is done in `momentuHMM`, where the same model only requires about 10 seconds to fit. Technical issues arising in the numerical maximisation, such as parameter constraints and numerical underflow, are fairly straightforward to deal with (Zucchini et al., 2016, pp. 50-54).

For complex models, local maxima of the likelihood function can become problematic (e.g. Myung, 2003). In this respect, it is generally advisable to try many different initial parameter vectors in the numerical optimisation, with initial values based on random draws, grid searches, or other methods (e.g. Brooks and Morgan, 1994; Biernacki et al., 2003; Schleer, 2015). With increasing model complexity, the issue of local maxima can be exacerbated to the extent that models can be practically non-identifiable due to effectively flat likelihood surfaces (e.g. Raue et al., 2009; Cole, 2019; Auger-Méthé et al., 2020).

The EM algorithm and MCMC approach to fitting HMMs are equally feasible approaches that can exploit the recursive schemes available for HMMs, such as the forward algorithm. In some cases, the EM algorithm can lead to a maximisation problem that is easier to solve than numerical maximisation of the likelihood, but EM can be more cumbersome to implement. The MCMC approach easily takes the HMM model into a Bayesian framework. However, MCMC samplers that include both the parameter vector ($\boldsymbol{\theta}$) and the latent states ($S_1, \ldots, S_T$) are inherently slow; sampling from the parameter vector only while using the forward algorithm to marginalise over states will often be preferable (Turek et al., 2016; Yackulic et al., 2020).

From a computational point of view, none of these methods is vastly superior to the others, but the appropriateness and efficiency of any given model fitting algorithm will be case dependent. There is no "free lunch" when it comes to model fitting, and no algorithm is guaranteed to converge to the global maximum (in case of maximum likelihood) or the posterior distribution (in case of MCMC sampling). Optimisation routines may require very long periods of time to locate the global maximum, and MCMC approaches may similarly have poor mixing and very slow convergence rates.

S4 Model selection & model checking

The task of selecting a particular HMM formulation from a suite of candidate models — which may differ, for example, in the number of underlying states, the family of distributions used for the state-dependent process, or the set of covariates that affect the state transition probabilities — is effectively analogous to any other model selection, say in regression analysis. As a consequence, general recommendations applicable also to such more established modelling classes directly transfer to the class of HMMs. In particular, model selection and checking can be approached in three steps:

1. consider both exploratory data analysis but also any relevant theory in order to keep the number of candidate models small (not just for computational reasons, but also to minimise the risk of a selection bias; cf. Zucchini, 2000);

2. use model selection criteria for guidance, but do not solely rely on such criteria (see Pohle et al., 2017, for a more detailed discussion of model selection in HMMs);

3. instead, carefully inspect the goodness-of-fit of any promising model to investigate if all patterns in the data pertinent to the study aim are sufficiently well captured by the model (and if not, to identify which components of the model may need to be modified).

S4.1 Model selection

For Step 1, in the VDBA example we initially restrict models to four or fewer states based on expert knowledge of the species' behaviour, and further on the observation that it is unlikely that any sensible behaviour classification would lead to five or more genuinely different behavioural modes in a one-hour period. Given this restriction, we can use model selection criteria (Step 2) such as the AIC or the BIC to select between models with up to four states. In this case, both the AIC and the BIC are minimised by the model with $N = 4$ states.

In general, when state characteristics are completely data-driven rather than pre-defined (e.g. Leos-Barajas et al., 2017), there is a tendency for HMMs with more states than seem biologically reasonable to be supported by standard model selection criteria such as AIC or BIC. The reason for this is that any additional state can, to some extent, compensate for any lack of structure or flexibility in the model formulation (cf. Pohle et al., 2017). In general, the judgements taken in Step 1 will often need to be relied upon to justify restricting the complexity of the model in spite of a poorer fit to the data.

S4.2 Model checking

Step 3 concerns model checking. Unfortunately, this important step is not as straightforward as for example in a regression analysis. The main *formal* approach to model checking in HMMs considers so-called *pseudo-residuals*, defined as

$$r_t = \Phi^{-1}(F_{X_t}(X_t)), \quad t = 1, \ldots, T,$$

where Φ is the cumulative distribution function of the standard normal distribution (such that Φ^{-1} is the corresponding quantile function) and

$$F_{X_t}(x) = \Pr(X_t \leq x \,|\, X_1 = x_1, \ldots X_{t-1} = x_{t-1}, X_{t+1} = x_{t+1}, \ldots X_T = x_T)$$

is the conditional cumulative distribution function of X_t, given all other observations, under the fitted model. The conditional distribution $F_{X_t}(x)$ can be obtained using a combination of the forward and the backward variables, respectively (for details, see Zucchini et al., 2016, pp. 82-83). For continuous variables it can be shown that *if the model is correct*, such that F_{X_t} is indeed the conditional cumulative distribution function of X_t given all other observations, then $F_{X_t}(X_t) \sim \text{Uniform}[0,1]$, such that $r_t \sim N(0,1)$. Thus, any deviation of the pseudo-residuals r_t

from normality indicates a potential lack of fit, which should be further investigated. While the r_t are not uncorrelated even for the correctly specified model, they should show much reduced correlation compared to the original sequence of observations. The main idea underlying these pseudo-residuals is that the distribution of each X_t within an HMM ought to be seen in the context of neighbouring observations in the sequence, which renders it non-trivial to assess which observations are extreme relative to the model. The transformation above, which is more generally known as the probability integral transform, yields a common scale, the standard normal distribution, for all observations, making it easier to identify observations that are extreme relative to the fitted model, and more generally any lack of fit.

For the VDBA example and the model with $N = 4$ states, Figure 3 shows the QQ-plot for the pseudo-residuals. They show deviation from normality in the tails of the distribution, indicating some lack of fit. In particular, the pattern in the residuals indicates that fewer observations of low accelerations were observed than one would expect under the fitted model. Nevertheless, the deviation is relatively minor and not necessarily a cause for concern, unless the focus of the analysis is specifically on these very small VDBA values and the associated behaviours.

Calculation and interpretation of the pseudo-residuals is somewhat tedious, as both the forward and the backward variables are required, and numerical underflow issues may need to be dealt with. Software packages that specialise in HMMs can be used to perform these calculations (see Section 4.1 in main text), but it can also be appealing to consider less technically involved approaches. An *informal* but often very useful approach to model checking consists of simulating observation sequences from the fitted model, then checking if relevant patterns in the real data are well replicated by the simulated data. The corresponding comparisons between simulated and real data could focus on summary statistics such as specific quantiles of the empirical distribution or the values of the sample autocorrelation function (ACF). For example, in the VDBA example, we simulated 100 datasets under the fitted model with $N = 4$

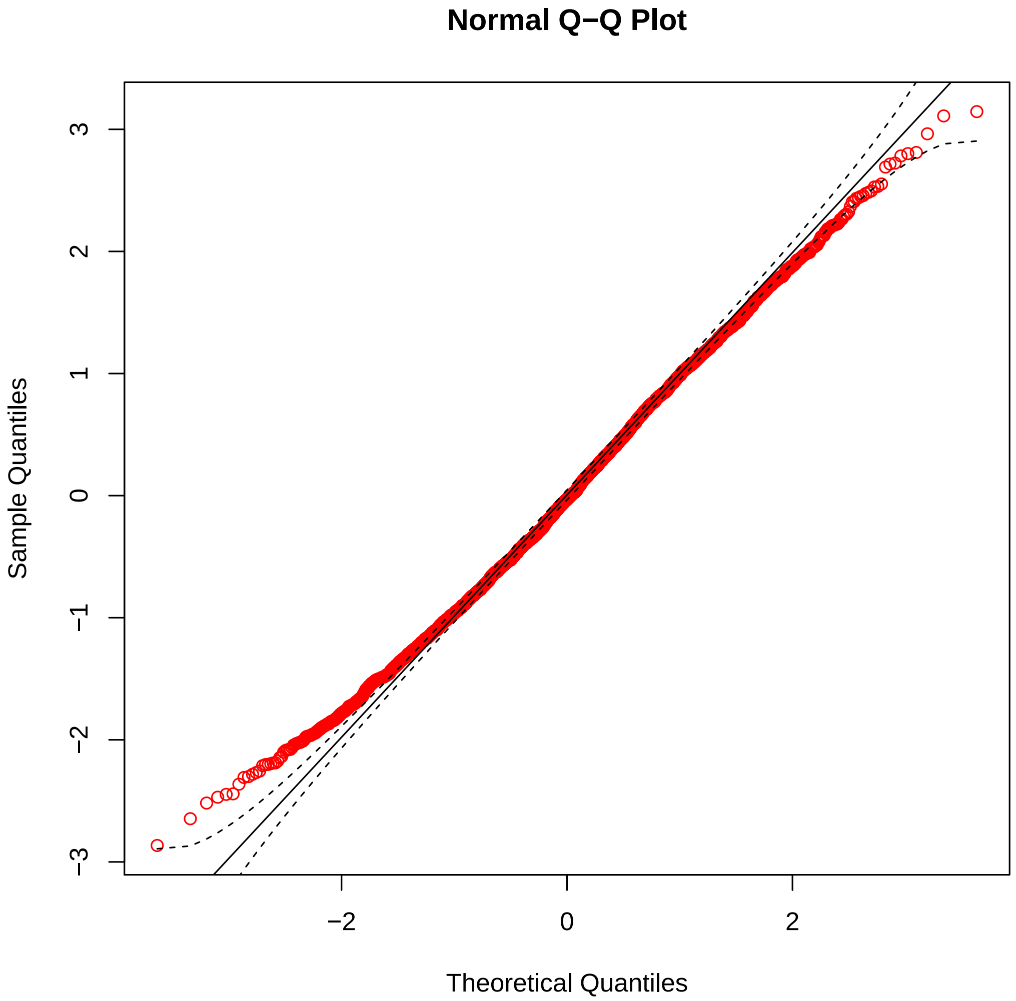

Figure 3: QQ-plot for the pseudo-residuals of the VDBA model with four states. Dashed lines indicate point-wise 95% confidence envelopes.

states. Figure 4 shows the quantile function and ACF estimated from these simulations. Overall, we find that the HMM fits the data fairly well, capturing the overall patterns in the data. However, we can also see the model's deficiencies: the quantile plot shows a minor lack of fit for very small observations, and the ACF shows that the correlation between neighbouring observations is higher than expected under the model, and that correlation at higher lags is also underestimated. The poorer fit of the ACF may indicate the limitation of the Markov assumption and the assumed geometric distribution in state dwell times, or alternatively a

violation of the conditional independence assumption of the observations. However, a misspecified model can also lead to correlation in the residuals even when these assumptions are true. There are many reasons why any given model might be incapable of explaining correlations in the data. Corresponding model extensions or alternative formulations could improve the fit — whether or not the corresponding effort necessary to fit those more complex models is warranted depends on the likely role of the lack of fit given the study aim.

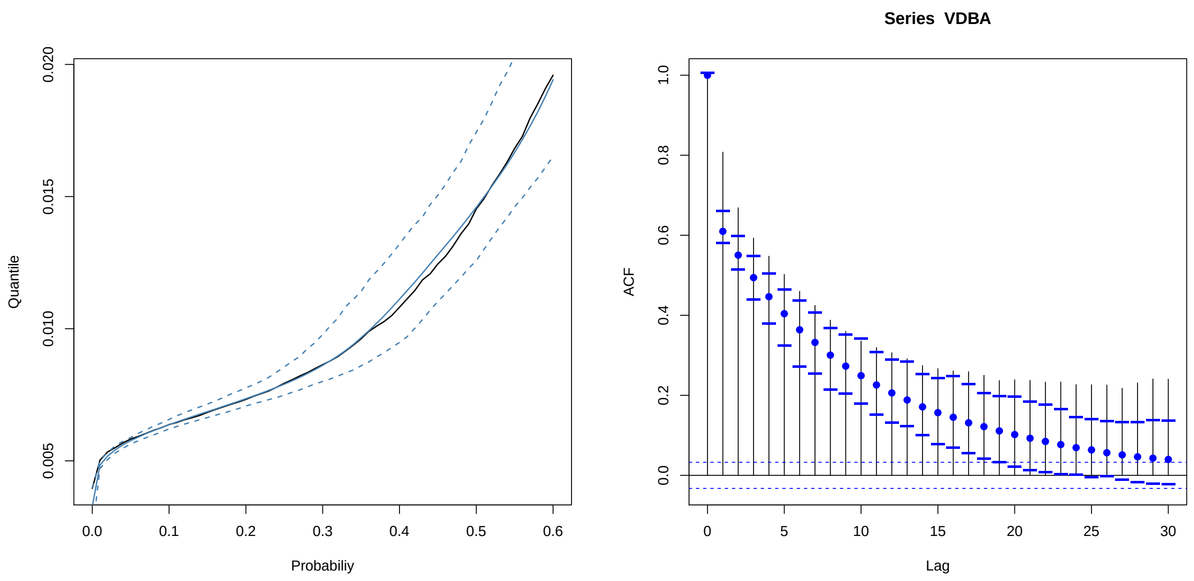

Figure 4: Estimated expected (blue) and empirical (black) quantile function (left) and autocorrelation function (right) using 100 simulated datasets from the VDBA model with four states; 95% quantile bounds are given for the expectations.

S5 State decoding

S5.1 Overview

Once parameters have been estimated and the model checking is complete, one goal may be to infer the hidden states $s_1, \dots, s_T$. This is called "state decoding". There are two approaches for decoding: local and global.

- **Local** state decoding is when we infer which value of s_t is *most likely for each time point separately*, that is, we seek the locally decoded state

$$l_t = \underset{s_t}{\operatorname{argmax}} \ \Pr(S_t = s_t \mid x_1, \ldots, x_T).$$

  Notice that the sequence of locally decoded states $l_1, \ldots, l_T$ ignores the serial dependence in the states: individually most likely does not mean they are jointly most likely.

- **Global** state decoding is when we infer which complete sequence of states is the *jointly most likely*, that is, we seek

$$(g_1, \ldots, g_T) = \underset{(s_1, \ldots, s_T)}{\operatorname{argmax}} \ \Pr(S_1 = s_1, \ldots, S_T = s_T \mid x_1, \ldots, x_T).$$

Whether to use locally or globally decoded states depends on the question one is trying to answer: whether one is interested in what state the Markov chain inhabits at a particular time or in the most likely sequence of states the chain followed. In practice the results from both approaches are usually similar. Figure 5 shows the globally decoded states for the VDBA example. In this case, the locally decoded states agreed with the globally decoded ones over 95% of the time.

S5.2 Computation

Local state decoding involves both the forward probabilities (defined in Section S3.2) and backward probabilities. Contrasting with the forward probabilities, $\alpha_t(j) = \Pr(x_1, \ldots, x_t, s_t = j)$, the backward probabilities are defined as $\beta_t(j) = \Pr(x_{t+1}, \ldots, x_T \mid s_t = j)$. The following useful relationship holds for any t:

$$\mathcal{L}(\boldsymbol{\theta} \mid x_1, \ldots, x_T) = \boldsymbol{\alpha}_t^T \boldsymbol{\beta}_t,$$

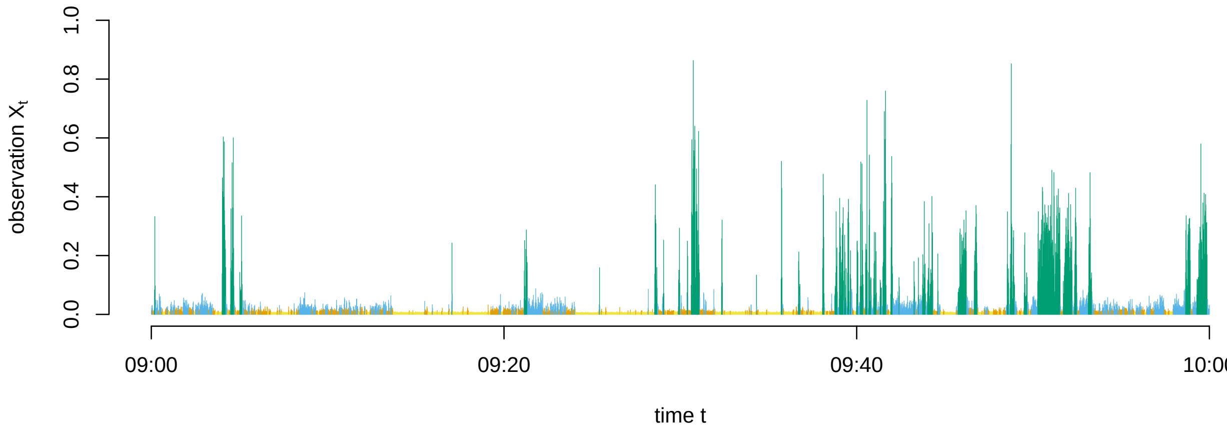

Figure 5: Sequence of vectorial dynamic body accelerations X_t, $t = 1, \dots, 3600$, colour-coded according to the most likely state sequence as inferred using the Viterbi algorithm.

since $\alpha_t(j)\beta_t(j) = \Pr(x_1, \dots, x_T, s_t = j)$. Local decoding involves maximising the quantity $\Pr(S_t = s_t \mid x_1, \dots, x_T)$ with respect to s_t, for every t. By basic probability laws, it can be shown that

$$\Pr(S_t = s_t \mid x_1, \dots, x_T) = \frac{\alpha_t(s_t)\beta_t(s_t)}{\mathcal{L}}. \tag{2}$$

At any time point t, this expression can be evaluated for $s_t = 1, \dots, N$, yielding the locally decoded state l_t as the argument maximising the expression. As with the forward algorithm, the forward-backward algorithm has computational complexity $\mathcal{O}(TN^2)$.

Global decoding is a more complex optimisation problem, but remarkably can be solved efficiently using the Viterbi algorithm. Let $q_t(j) = \Pr(s_1 = g_1, \dots, s_{t-1} = g_{t-1}, s_t = j, x_1, \dots, x_T)$, that is, suppose we know the optimal sequence up to time $t-1$ and so $q_t(j)$ is the joint probability (with the data) that the state at time t is j. The Viterbi algorithm makes use of the following recurrence: $q_t(j) = \max_i q_{t-1}(i)\gamma_{i,j} f(x_t \,|s_t = j)$. Clearly $q_1(j) = \delta_j f(x_1 \mid s_1 = j)$ and so q_t can be computed for every t. The globally decoded states can then be determined by going backwards in time: choose $g_T = \text{argmax}_j\, q_T(j)$ and then $g_t = \text{argmax}_j\, q_t(j)\gamma_{j,g_{t+1}}$ for $t < T$. The Viterbi algorithm therefore also has computational complexity $\mathcal{O}(TN^2)$.

It is worth noting that only local decoding via Eq. (2) provides an uncertainty quantification

with respect to the decoded states, i.e. probability statements on which state may have been active at any time point. Global decoding via the Viterbi algorithm merely provides the hard decoded most likely state sequence without any probabilistic information on potential state misclassifications.

S6 Interpretation

We have formulated the HMM, estimated the parameters, selected between candidate models, checked how well the model fits, inferred the latent states, and now must interpret the final results. It is important to remember at this stage two things:

- Even though the state process may have been formulated according to certain known (or hypothesised) properties of an underlying ecological process, the parameters of the state-dependent distributions and the transition probability matrix are driven by the data and so may instead lead to states that do not correspond to our original intentions. Our choice of the number of states, the data streams to include, and the structure of the HMM components can encourage the model to reflect the ecological dynamics we are interested in, but this is not formally guaranteed and so results must be interpreted with caution. HMMs are aids to our understanding of these ecological processes, but not substitutes for ecological theory.

- The results obtained must be interpreted in light of the assumptions that have been made. The number of assumed states, the assumed form of the state-dependent distributions, the Markov assumption, and the conditional independence assumption. Whether or not these assumptions are well enough respected in any given application must be questioned during model checking, and any deficiencies taken into account when interpreting results. Identifying important issues with an HMM may lead to specifying a more complex model (e.g. extensions of HMMs as discussed in Section 2.3 of the main

text), or could lead to restricting research conclusions to robust, but weak statements. When the assumptions of HMMs and their extensions fail to adequately describe the underlying ecological dynamics of interest, then other forms of latent variable models may be more appropriate (see Box 1 in main text).

For the VDBA example, we settled on an HMM with $N = 4$ states. Considering the estimated state-dependent parameters in the context of ecological knowledge available for this species, it is possible for experts to qualitatively associate these states with behavioural modes: "resting", "minimal activity" (e.g. preening), "moderate activity" (e.g. walking, digging), and "flying", which based on global state decoding were respectively assigned to 31%, 27%, 27%, and 14% of time steps over a period of one hour. In model checking, we found some relatively minor issues with both the marginal distribution and the dependence structure. If our interest is in the broad classification of behaviours and the observations that relate to these behaviours, these issues may not be important. However, if for example our research question concerns how long individuals spend in each state, then the model's failure to capture the full empirical dependence structure may need to be addressed with more sophisticated HMMs or alternative modelling frameworks.

Overall, this tutorial briefly demonstrates the workflow when trying to uncover underlying ecological dynamics with an HMM. Like all modelling, it comes with assumptions and limitations, but HMMs also bring computational efficiency and a structure that has been found to be applicable to an impressively wide range of ecological applications.

References

Auger-Méthé, M., Newman, K., Cole, D., Empacher, F., Gryba, R., King, A. A., Leos-Barajas, V., Flemming, J. M., Nielsen, A., Petris, G., et al. (2020). An introduction to state-space modeling of ecological time series. *arXiv preprint arXiv:2002.02001.*

Biernacki, C., Celeux, G., and Govaert, G. (2003). Choosing starting values for the em algorithm for getting the highest likelihood in multivariate gaussian mixture models. *Computational Statistics & Data Analysis*, 41(3-4):561–575.

Brooks, S. P. and Morgan, B. J. (1994). Automatic starting point selection for function optimization. *Statistics and Computing*, 4(3):173–177.

Cole, D. J. (2019). Parameter redundancy and identifiability in hidden Markov models. *METRON*, 77(2):105–118.

Ellison, A. M. (2004). Bayesian inference in ecology. *Ecology Letters*, 7(6):509–520.

Fahlbusch, J. A. and Harrington, K. J. (2019). A low-cost, open-source inertial movement GPS logger for eco-physiology applications. *Journal of Experimental Biology*, 222(23):jeb211136.

Leos-Barajas, V., Photopoulou, T., Langrock, R., Patterson, T. A., Watanabe, Y. Y., Murgatroyd, M., and Papastamatiou, Y. P. (2017). Analysis of animal accelerometer data using hidden Markov models. *Methods in Ecology and Evolution*, 8(2):161–173.

Li, M. and Bolker, B. M. (2017). Incorporating periodic variability in hidden markov models for animal movement. *Movement ecology*, 5(1):1.

MacKenzie, D. I., Nichols, J. D., Lachman, G. B., Droege, S., Andrew Royle, J., and Langtimm, C. A. (2002). Estimating site occupancy rates when detection probabilities are less than one. *Ecology*, 83(8):2248–2255.

McClintock, B. T. and Michelot, T. (2018). momentuHMM: R package for generalized hidden Markov models of animal movement. *Methods in Ecology and Evolution*, 9(6):1518–1530.

Morales, J. M., Haydon, D. T., Frair, J., Holsinger, K. E., and Fryxell, J. M. (2004). Extracting more out of relocation data: building movement models as mixtures of random walks. *Ecology*, 85(9):2436–2445.

Myung, I. J. (2003). Tutorial on maximum likelihood estimation. *Journal of Mathematical Psychology*, 47(1):90–100.

Newman, K. B., Buckland, S. T., Morgan, B. J. T., King, R., Borchers, D. L., Cole, D. J., Besbeas, P., Gimenez, O., and Thomas, L. (2014). *Modelling population dynamics: model formulation, fitting and assessment using state-space methods*. Springer.

Patterson, T. A., Basson, M., Bravington, M. V., and Gunn, J. S. (2009). Classifying movement behaviour in relation to environmental conditions using hidden Markov models. *Journal of Animal Ecology*, 78(6):1113–1123.

Patterson, T. A., Parton, A., Langrock, R., Blackwell, P. G., Thomas, L., and King, R. (2017). Statistical modelling of individual animal movement: an overview of key methods and a discussion of practical challenges. *AStA Advances in Statistical Analysis*, 101(4):399–438.

Pohle, J., Langrock, R., van Beest, F. M., and Schmidt, N. M. (2017). Selecting the number of states in hidden Markov models: pragmatic solutions illustrated using animal movement. *Journal of Agricultural, Biological and Environmental Statistics*, 22(3):270–293.

Qasem, L., Cardew, A., Wilson, A., Griffiths, I., Halsey, L. G., Shepard, E. L., Gleiss, A. C., and Wilson, R. (2012). Tri-axial dynamic acceleration as a proxy for animal energy expenditure; should we be summing values or calculating the vector? *PLoS ONE*, 7(2).

R Core Team (2019). *R: A Language and Environment for Statistical Computing*. R Foundation for Statistical Computing, Vienna, Austria.

Rabiner, L. R. (1989). A tutorial on hidden Markov models and selected applications in speech recognition. *Proceedings of the IEEE*, 77(2):257–286.

Raue, A., Kreutz, C., Maiwald, T., Bachmann, J., Schilling, M., Klingmüller, U., and Timmer,

J. (2009). Structural and practical identifiability analysis of partially observed dynamical models by exploiting the profile likelihood. *Bioinformatics*, 25(15):1923–1929.

Schleer, F. (2015). Finding starting-values for the estimation of vector star models. *Econometrics*, 3(1):65–90.

Turek, D., de Valpine, P., and Paciorek, C. J. (2016). Efficient Markov chain Monte Carlo sampling for hierarchical hidden Markov models. *Environmental and Ecological Statistics*, 23(4):549–564.

White, G. C. and Burnham, K. P. (1999). Program MARK: Survival estimation from populations of marked animals. *Bird Study*, 46:S120–S138.

Yackulic, C. B., Dodrill, M., Dzul, M., Sanderlin, J. S., and Reid, J. A. (2020). A need for speed in Bayesian population models: a practical guide to marginalizing and recovering discrete latent states. *Ecological Applications*, 30:e02112.

Zucchini, W. (2000). An introduction to model selection. *Journal of mathematical psychology*, 44(1):41–61.

Zucchini, W., MacDonald, I. L., and Langrock, R. (2016). *Hidden Markov Models for Time Series: An Introduction Using R.* CRC Press, second edition.
